# Sensor Placement Optimization for Shape Sensing of Plates and Shells Using Genetic Algorithm and Inverse Finite Element Method

**DOI:** 10.3390/s22239252

**Published:** 2022-11-28

**Authors:** Maryam Ghasemzadeh, Adnan Kefal

**Affiliations:** 1 Composite Technologies Center of Excellence, Istanbul Technology Development Zone, Sabanci University-Kordsa Global, 34906 Istanbul, Turkey; 2 Integrated Manufacturing Technologies Research and Application Center, Sabanci University, 34956 Istanbul, Turkey; 3 Faculty of Engineering and Natural Sciences, Sabanci University, 34956 Istanbul, Turkey

**Keywords:** inverse finite element method, genetic algorithm, optimal sensor placement, deformation monitoring, structural health monitoring

## Abstract

This paper reports the first investigation of the inverse finite element method (iFEM) coupled with the genetic algorithm (GA) to optimize sensor placement models of plate/shell structures for their real-time and full-field deformation reconstruction. The primary goal was to reduce the number of sensors in the iFEM models while maintaining the high accuracy of the displacement results. Here, GA was combined with the four-node quadrilateral inverse-shell elements (iQS4) as the genes inherited through generations to define the optimum positions of a specified number of sensors. Initially, displacement monitoring of various plates with different boundary conditions under concentrated and distributed static/dynamic loads was conducted to investigate the performance of the coupled iFEM-GA method. One of these case studies was repeated for different initial populations and densities of sensors to evaluate their influence on the accuracy of the results. The results of the iFEM-GA algorithm indicate that an adequate number of individuals is essential to be assigned as the initial population during the optimization process to ensure diversity for the reproduction of the optimized sensor placement models and prevent the local optimum. In addition, practical optimization constraints were applied for each plate case study to demonstrate the realistic applicability of the implemented method by placing the available sensors at feasible sites. The iFEM-GA method’s capability in structural dynamics was also investigated by shape sensing the plate subjected to different dynamic loadings. Furthermore, a clamped stiffened plate and a curved shell were also considered to assess the applicability of the proposed method for the shape sensing of complex structures. Remarkably, the outcomes of the iFEM-GA approach with the reduced number of sensors agreed well with those of the full-sensor counterpart for all of the plate/shell case studies. Hence, this study reveals the superior performance of the iFEM-GA method as a viable sensor placement strategy for the accurate shape sensing of engineering structures with only a few sensors.

## 1. Introduction

Mechanical structures are prone to various defects and faults that can rapidly grow and lead to tragic failures. Numerous accidents causing human and environmental loss have been annually reported due to structural anomalies and deficiencies in aerospace vehicles, civil platforms, and ship/marine structures [1,2,3]. The main portion of these faults is associated with the reduction in the overall structural integrity; therefore, the practice to determine a structure’s real-time information regarding its global or local state, known as structural health monitoring (SHM), plays a vital role in the repair and maintenance of structures [4,5,6,7].

SHM is a sensor-integrated procedure that processes the data collected from a sensing system to detect the defects triggered under operational conditions. SHM consists of five keys: (i) sensing system description, (ii) sensor data acquisition, (iii) shape/strain/stress-sensing process, (iv) global/local failure prediction, and (v) decision-making process. Real-time deformation evaluation [8,9], vibration-based monitoring [10,11,12,13], and other SHM approaches [14,15] involve the implementation of sensors such as accelerometers, displacement sensors, or strain gauges [16,17,18], which are directly mounted on structural components to measure mechanical responses. SHM can assess the structural integrity, cracks, and damage during their initial phase and subsequently reduce the maintenance costs. The implementation of SHM for the real-time reconstruction of full-field displacements of the structure, known as shape sensing, has grown as emerging method in recent research efforts [19,20,21,22,23].

The Inverse finite element (iFEM) method, a novel approach of SHM proposed by Tessler and Spangler [24], originally employed a three-node inverse-shell element (iMIN3) for the shape sensing of plate structures. iFEM is a strain/displacement based SHM technique which, in contrast to other available SHM methods, is suitable to monitor any displacement of complex topologies and stress fields with intricate boundary conditions by using a network of in situ strain sensors and measured strains [25,26,27,28,29]. The advantages of iFEM have recently drawn significant attention since various scientists have attempted to improve the available iFEM equations to achieve better results in the last decade. For example, the seminal work of Kefal et al. [30] is worth highlighting herein, which increased the practical utility of iFEM by developing a novel inverse-shell element, iQS4. This element includes hierarchical drilling rotation degrees-of-freedom (DOF) to extend the suitability of the method for the shape sensing of large-scale structures. Moreover, the iQS4 element has the advantage of avoiding singular responses when simulating complex shell structures. Furthermore, features of its formulation can prevent membrane and shear locking phenomena. These benefits have motivated many other investigations conducted by employing iQS4/iFEM methodology in the last few years. As an example, Kefal et al. applied iQS4 elements to reconstruct the displacement field of a chemical tanker [31], a container ship [32], and a bulk carrier [33] from discrete strain sensors and subsequently obtained their full-field strains and stresses in real-time. The experimental strains were simulated with high-fidelity finite element method (FEM) analysis where hydrodynamic loads of the large-scale structures for a certain wave frequency were calculated with an in-house panel-method code [34]. Li et al. [35] implemented iFEM/iQS4 to analyze the offshore wind turbine tower structure under static and dynamic loading conditions; they attempted to reconstruct the full-field displacement by considering the gravity of the upper frame and tower for the static loading and aerodynamic forces for the dynamic cases. Moreover, Espesito et al. [36] applied iFEM/iQS4 methodology to monitor the displacement of a wing box model and compared its shape-sensing capability with other methods (e.g., modal method [37] and Ko’s displacements theory [38]). This study proved that the iQS4 element provides superior displacement accuracy while requiring a slightly more demanding number of sensors than the other methods for shape sensing.

With the recent development of composite materials and their application in different structures, monitoring their structural integrity and safety has become an important topic of interest in the field of SHM [39,40,41]. In this regard, many researchers have employed iFEM to perform deformation reconstruction, strain sensing, and the stress monitoring of composite structures [42,43,44,45]. These studies include applications of iFEM to at/curved composite structures with thin/thick or moderately thick lamination regimes. Most importantly, Kefal et al. [45] enhanced the available iFEM formulation [44] by incorporating the full kinematic relations of the refined zigzag theory [46] with the iFEM least-squares formulation. The enhanced iFEM method [45] provided highly accurate stress and strain distribution results through the thickness of composite laminates using a few strain sensors. Apart from composite structures, Gherlone et al. [47] attempted to estimate the frame and beam deformation using iFEM based on the kinematic relations of Timoshenko’s theory. Moreover, Roy et al. [48] reformulated this inverse method for the shape sensing of beams with complex cross-sections. These involved specific fixed coefficients and functions for identifying the shear strain variation in any cross-section and evaluating its equivalent value at its centroid. Perusing improvement of the results of analyzing structures with iFEM, Kefal et al. [49] offered the coupling of isogeometric analysis (IGA) with the iFEM methodology for the shape sensing of complex/thin shell structures. The isogeometric iFEM approach aims to provide highly accurate small/large-deformation shape reconstruction of the doubly curved shell structures with a lower number of sensors. Furthermore, Papa et al. [50] took advantage of iFEM for real-time full-field displacement reconstruction of a UAS wing box with the utilization of a triangular shape at the shell element, whereas Kefal et al. [51] promoted the model of iFEM combined with RZT to shape sense airplane-wing-shaped plates under different types of loading. These research efforts extracted the strain data from sensors with various densities and alignments, and then compared its results with the computational or experimental methods. Development in sensor technology and the evolution in the current sensor products (e.g., suggesting fiber Bragg grating (FBG) sensors) have enabled the exploitation of many sensors; however, due to the weight restriction for many structures and the cost of instrumentation, the number of applicable sensors is limited. Regarding the importance of this issue, many researchers have focused on exercising the location and alignment of the sensors to achieve the most accurate data by using the minimum possible number of sensors. For this goal, some studies have been conducted to determine the optimum sensor placement [52,53,54,55,56,57]. One of the highlighted research studies in this field [52,53] examined the optimum arrangement of sensors by minimizing the norm of the Fisher information matrix, which was obtained from the modal and measurement of the covariance matrix. In addition, Salama [54] suggested implementing the modal kinetic energy as a criterion to select the eligible sensors to achieve an optimum arrangement.

Despite the available literature on the optimum sensor placement in vibration-based structural health monitoring, the lack of a comprehensive method to define the optimum placement in strain-based SHM is evident. The primary strategy of the inverse finite element method is to collect data from the strain rosette sensors and employ it through the iFEM formulation to regenerate the real-time full-field deformation [58,59,60,61,62,63]; thus, the location of these strain gauges plays an essential role in the certainty of the results. In this regard, Kefal et al. [58], in the endeavor of introducing a new inverse eight-node element (iCS8) to shape sensing curved structural components of marine structures, investigated the effect of the sensor sparsity and its effect on the accuracy of the results. In another effort, Abdollahzadeh et al. [59] reduced the number of sensors and changed full-sensor cases with reduced cases to examine the effect of sensors on the accuracy of the results; in this research, the author improved the accuracy of reduced sensor cases by pre-extrapolated strain measurements. Kefal et al. [60] employed isogeometric iFEM analysis for shell structures to improve the displacement results of coarse mesh with a few sensors compared to the results of high-fidelity FEM analysis. Roy et al. [61] struggled to suggest a proper position for sensors on a rectangular plate. They explored various sensor patterns, taking a specified volume fraction for sensors and implemented them in such locations to achieve the best result and the minimum displacement error.

As above-mentioned, most of the research conducted to examine the best place for sensors by leveraging iFEM methodology have estimated the critical locations for available sensors by examining experience-based-selected/specific positions and the trial and error process. However, this process is not always practical for complex structural topologies and mixed boundary conditions. Thus, to scrutinize the role of sensor placement based on iFEM, the absence of an automatic approach to define the best spots for sensors is evident. This key gap is filled with the objectives of the present study by attempting to reduce the number of sensors in the iFEM method while maintaining the accuracy of the results. The primary and novel aim of the current work is to couple the genetic algorithm (GA) optimization method with iFEM to determine the best locations for a given number of sensors. To the best of our knowledge, this was the first study that merged iFEM-GA methods to optimize the sensor placement model of the plate/shell structures for the purpose of shape sensing in the literature.

The rest of this paper is structured as follows. First, the theoretical basis of iFEM and the implementation of iFEM-GA is introduced. Then, the number of efficient initial populations in GA is examined to avoid local optimization, premature convergence, and ensure the proper diversity to ascertain the most eligible parents for the next generation. After this, as a practical application, a clamped plate with different loading conditions was considered to investigate the optimum locations for the available sensors with and without geometrical constraints. Moreover, the dynamic response of the plate subjected to time-dependent loadings with different frequencies was reconstructed using the proposed method to capture the different vibrational modes. The obtained results for all cases were compared with high-fidelity FEM and full-sensor placement models of the iFEM analysis. Afterward, a stiffened panel was considered to search for the best locations for the sensors to investigate the applicability of the introduced method in reconstructing the deformation of the complex geometries. Next, the proposed method was implemented to search for the optimum positions for the sensors in curved shell structures to the real-time reconstruction of its displacement. Finally, several closing remarks regarding the scientific advances of the iFEM-GA algorithm are presented in the concluding section.

## 2. Mathematical Formulation of iFEM-GA Method

### 2.1. The iQS4 Inverse-Shell Element

The four-node quadrilateral inverse-shell element, iQS4 [30], was implemented in this research study to examine the performance of the iFEM-GA method. The iQS4 element was considered with a uniform thickness of 2h. Node coordinates were defined according to the local coordinate system of (x,y,z) located at the centroid of the mid-plane (z=0) of the element. Here, (x,y) and z∈[−h,h] describe the in-plane and thickness coordinates, respectively. Kinematic relations were established according to the first-order shear deformation theory. The membrane displacements were interpolated using in-plane translational and drilling rotational degrees of freedom (DOF) whereas deflection and counterclockwise bending rotations were defined by out-of-plane translational and in-plane rotational DOF according to [30]. Taking relevant first-order derivatives of the kinematic variables, the normal and shear strains of the iQS4 element can be determined as:

(1a) $$\left\{\begin{array}{c} \varepsilon_{xx}\\ \varepsilon_{yy}\\ \gamma_{xy} \end{array}\right\}\equiv e(u^{e})+z\kappa (u^{e})=B^{m}u^{e}+zB^{b}u^{e}$$

(1b) $$\left\{\begin{array}{c} \gamma_{xz}\\ \gamma_{yz} \end{array}\right\}\equiv g(u^{e})=B^{s}u^{e}$$

where the symbols of e, κ, g, and $u^{e}$ represent the membrane strains, bending curvatures, transverse-shear strains, and nodal DOF of the iQS4 element, respectively. Moreover, the $B^{m}$ matrix contains the corresponding derivatives of shape functions that construct the membrane strain response of the element. Furthermore, $B^{b}$ and $B^{s}$ hold the derivatives of shape functions that define the element bending response. The explicit forms of these matrices can be found in [30].

**Figure 1:**
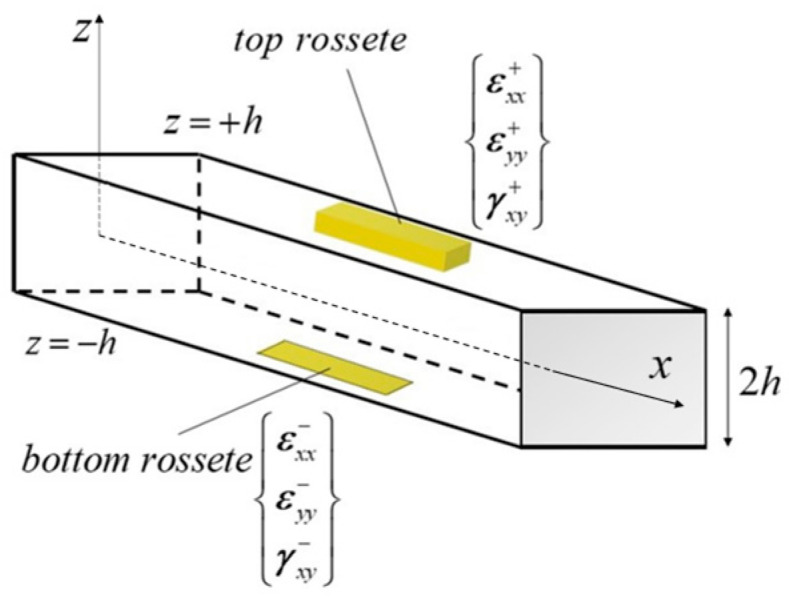
Discrete surface strains measured by strain rosettes within the iQS4 element.

The input for iFEM formulation is the discrete strain data collected from on-board sensors such as conventional strain rosette or embedded fiber-optic sensor (fiber Bragg grating, FBG) networks. These data have a profound effect on the accuracy of the obtained results. To experimentally compute the membrane section strain $e^{\varepsilon }$ and bending curvature $\kappa^{\varepsilon }$ corresponding to the analytical counterparts (as given in Equation (1a)), the in situ strain rosettes need to be mounted at the top and bottom surface of the plate (iQS4 model), as depicted in Figure 1.

The measured strain data obtained from discrete n locations within element $\left(x_{\alpha }=x_{\alpha },x_{\alpha },\pm h\right)$ were utilized to express the discrete values of membrane strain $e_{\alpha }^{\varepsilon }$ and bending curvature $\kappa_{\alpha }^{\varepsilon }$ as:

(2a) $$e_{\alpha }^{\varepsilon }=\frac{1}{2}{\left\{\begin{array}{c} \varepsilon_{xx}^{+}+\varepsilon_{xx}^{-}\\ \varepsilon_{yy}^{+}+\varepsilon_{yy}^{-}\\ \gamma_{xy}^{+}+\gamma_{xy}^{-} \end{array}\right\}}_{\alpha }\;\;\;\;\;\;\;\;\;\;(\alpha =1,n)$$

(2b) $$\kappa_{\alpha }^{\varepsilon }=\frac{1}{2h}{\left\{\begin{array}{c} \varepsilon_{xx}^{+}-\varepsilon_{xx}^{-}\\ \varepsilon_{yy}^{+}-\varepsilon_{yy}^{-}\\ \gamma_{xy}^{+}-\gamma_{xy}^{-} \end{array}\right\}}_{\alpha }\;\;\;\;\;\;\;\;\;\;(\alpha =1,n)$$

One can use these discrete values of the experimental section strains in smoothing procedures [28] to obtain their continuous form within the element, $e_{\alpha }^{\varepsilon }\to e^{\varepsilon }$ and $\kappa_{\alpha }^{\varepsilon }\to \kappa^{\varepsilon }$. If a single set of in situ section strains is available per iQS4 element, namely n→1, the continuous form of the section strains, $e^{\varepsilon }$ and $\kappa^{\varepsilon }$ can be assumed to be uniformly distributed (spatially constant) within the element. This reasonable assumption is often desirable for a practical and low-cost sensor placement model. It is also always valid since an individual element domain has an infinitesimally small surface compared to the whole surface of the structure being monitored. For such a sensor placement configuration, the centroid of the element can be chosen to mount the top and bottom strain rosettes on the structure to experimentally obtain the average values of $e^{\varepsilon }$ and $\kappa^{\varepsilon }$ over the iQS4 element [62,63].

The iFEM methodology minimizes a weighted least-squares function of the experimental and numerical section strains with respect to the nodal DOF of the full discretization to perform shape sensing of the structure using the discretized model and sensor data. This function is carefully defined for an individual iQS4 element as:

(3) $$\Phi_{e}(u^{e})=\frac{1}{A_{e}}\iint_{A_{e}}(w_{e}{\left\|e(u^{e})-e^{\varepsilon }\right\|}^{2}+w_{k}{\left(2h\right)}^{2}{\left\|\kappa (u^{e})-\kappa^{\varepsilon }\right\|}^{2}+w_{g}{\left\|g(u^{e})-g^{\varepsilon }\right\|}^{2})dxdy$$

In the above equation, $g(u^{e})$ corresponds to the transverse shear strain, and $g^{\varepsilon }$ stands for the experimentally collected transverse shear strain. In the deformation of plate/shell structures, the contribution of transverse shear is much smaller than that of the bending curvature; thus, this term can be safely omitted. On the other hand, the weighting constant of $w_{e},w_{k}$, and $w_{g}$ are positive values associated with the section strains, controlling the consistency between the experimentally measured data and analytical section strains.

In the general form of the inverse finite element method, all of the inverse elements contain the sensor; however, this study attempted to obtain the result using a reduced number of sensors. To this end, weighting constants play a significant role in conducting accurate deformation reconstruction. In the case of the existing measured in situ measures, the weighting constants are set to unity; for the missing strain component values, the corresponding weighting constants are assigned to a very small number (e.g., $\beta =10^{-4}$). To maintain the accuracy of the results, the location of the strain-less elements, expressly the place of available sensors, is crucial. Note that since there is no experimental measurement following shear stress, its conjugated constant value was set to a very small number $w_{k}=10^{-4}$. Rewriting the Euclidean norms of Equation (3) in terms of the $B^{i}(i=m,b,s)$ matrices and subsequently minimizing the $\Phi_{e}(u^{e})$ for the nodal DOF yields the following form [30]:

(4) $$\frac{\partial \Phi_{e}(u^{e})}{\partial u^{e}}=k^{e}u^{e}-f^{e}=0$$

In Equation (4), $k^{e}$ is the element shape matrix, which can be written in terms of the $B^{i}(i=m,b,s)$ matrices as:

(5) $$k^{e}=\frac{1}{A_{e}}\iint_{A_{e}}(w_{e}{\left(B^{m}\right)}^{T}B^{m}+w_{k}{\left(2h\right)}^{2}{\left(B^{b}\right)}^{T}B^{b}+w_{g}{\left(B^{s}\right)}^{T}B^{s})dxdy$$

where $f^{e}$ is the right-hand-side vector, which can be defined utilizing the data collected from the sensors as:

(6) $$f^{e}=\frac{1}{A_{e}}\iint_{A_{e}}(w_{e}{\left(B^{m}\right)}^{T}e^{\varepsilon }+w_{k}{\left(2h\right)}^{2}{\left(B^{b}\right)}^{T}\kappa^{\varepsilon }+w_{g}{\left(B^{s}\right)}^{T}B^{s})dxdy$$

The surface area integrals in Equations (5) and (6) can be numerically computed for the iQS4 element by employing the Gaussian quadrature method with 2×2 integration points. Specifically, if a single set of top–bottom strain rosettes are available for the iQS4 element, the experimental section strains can be assigned identically (as per the assumption of the spatially uniform distribution elaborated above) for all Gaussian points during the numerical integration in Equation (6). Accordingly, during the optimization process detailed in Section 2.2, we considered that each iQS4 element can include either a maximum of one set of sensor data or no sensor measurements to obtain the most practical and low-cost sensor placement models.

Ultimately, for the entire discretization, the formulation derived for an element can be extended to the global linear equation through an assembly process (i.e., based on standard finite element procedures):

(7a) KU=F

(7b) $$K=\sum_{e=1}^{nel}{\left(T^{e}\right)}^{T}k^{e}T^{e},\;F=\sum_{e=1}^{nel}{\left(T^{e}\right)}^{T}f^{e},\;U=\sum_{e=1}^{nel}{\left(T^{e}\right)}^{T}u^{e}$$

where the summation symbol represents the classical finite element assembly process; K, U, and F are the assembled shape matrix, global nodal displacement, and global right-hand-side (experimental strain) vector, respectively; and $T^{e}$ is the transformation matrix of the nodal DOFs of the iQS4 element from the local to the global coordinate system. This equation system can be solved by (1) applying a problem-specific constraint condition to the K matrix, $K\to K_{R}$; (2) taking its inverse, $K_{R}^{-1}$; and (3) multiplying the resultant inverse matrix with a reduced form of the global experimental strain vector, $F_{R}$. As a result, the displacement DOF of all nodes, representing the global deformed shape of the full discretization, can be obtained from this solution.

### 2.2. Coupling iFEM and Genetic Algorithm

Weighting constants in Equations (5) and (6) are the keys to link the iFEM formulation and genetic algorithm. The values of $w_{e}=w_{k}=1$ are associated with the existence of a sensor within an element and can be assigned to the desired number of the inverse elements concerning the specified sensor volume fraction. Moreover, these weighting values enable us to remove sensors from some elements through strain-less inverse element analysis. For this type of measurement, all weighting factors are set to a very small value, $w_{e}=w_{k}=w_{g}=10^{-4}$. GA is a heuristic optimization algorithm based on the natural selection of species. It works based on defining the initial population, reproduction, mutation of the gene band, and natural selection to reach the most favorable population. In this optimization method, genes carrying information of the problem form a string known as individuals (chromosomes), where a defined number of these individuals constitutes an initial population. According to Darwin’s theory, individuals struggle for existence, and their chance to survive depends on their genes accommodating to the environment. In this paper, the binary coding method was adopted to assign values to the genes [64]. The number of genes was set according to the number of inverse elements employed in iFEM discretization. To allocate $n_{total}$ sensors in $n_{nel}$ elements, a string with nel degrees of freedom that represents an individual in the genetic algorithm is considered. Accordingly, assigning 1 to each gene is interpreted as placing a sensor in the corresponding element, and 0 means the iFEM element without a sensor is:

(8) $$\tau_{e}=\{\begin{array}{c} 1\;\;\;iQS4\;\;\;element\;e\;with\;sensor\\ 0\;\;\;iQS4\;\;\;element\;e\;without\;sensor \end{array}\;\;\;\;\;\;\;\;\;\;\;\;\;\;\;\;\;\;(e=1,2,3,\ldots ,nel)$$

The genetic algorithm sets sensors in random locations to shape individuals (chromosomes) of the initial population. Each of the chromosomes needs to meet the constraints of the optimization problem, which are the number of available sensors, $n_{total}$, and feasibility of the selected locations, in this study. Table 1 concisely illustrates the above explanation with λ individuals randomly assigned a value to their genes, which were considered as the initial population of the optimization problem.

**Figure 2:**
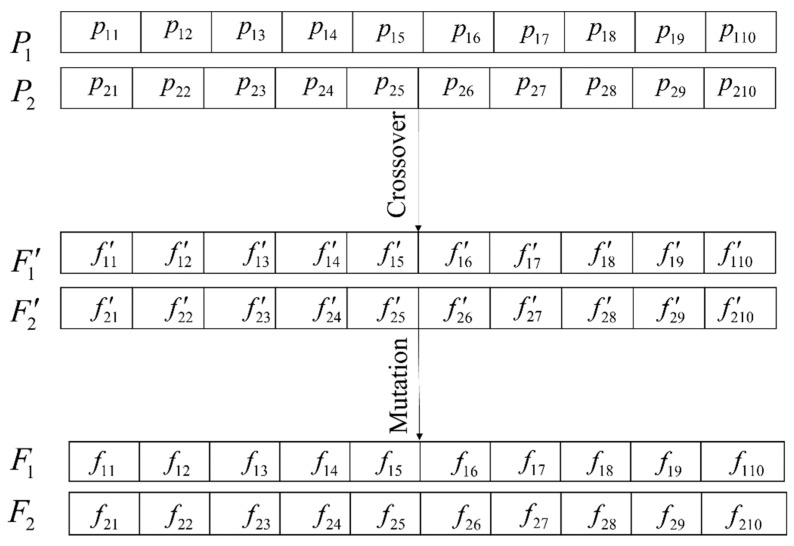
Generating new offspring using the genetic algorithm.

The next step is selecting the most eligible parents to produce the next generation. GA ranks the individuals based on the cost function and picks the most qualified couples to have their offspring. Each couple of parents mix their genes based on a crossover system to perform their next generations. Crossover is an operator that exchanges the parents’ genes to generate offspring. There are several crossover techniques, and uniform crossover was chosen in this paper [64]. Assume the case that two strings of $P_{1}=p_{11},p_{12},p_{13},\ldots ,p_{1e}$ and $P_{2}=p_{21},p_{22},p_{23},\ldots ,p_{2e}$ are the chromosomes corresponding to the parents and $F_{1}=f_{11},f_{12},p_{13},\ldots ,f_{1e}$ and $F_{2}=f_{21},f_{22},p_{23},\ldots ,f_{2e}$ are the mutated descendants. Equation (9a,b) state the relation between the parent and offspring genes based on uniform crossover as:

(9a) $$f_{1e}^{\prime }=\gamma p_{1e}+(1-\gamma )p_{1e}$$

(9b) $$f_{2e}^{\prime }=\gamma p_{2e}+(1-\gamma )p_{2e}$$

where γ is a random binary number that specifies the correlation of each parent’s gene in the corresponding offspring gene. Since the crossover step randomly exercises the gene value, the obtained genes of offspring $f_{1e}^{\prime }$ and $f_{2e}^{\prime }$ may not fulfill the condition of the optimization problem (immature offspring). Therefore, these generations undergo the mutation process where the sensors are randomly added or dismissed to achieve the desired offspring (mutated offspring). Note that controlling the population heterogeneity is very important to search the global optima and to avoid premature convergence [64]; thus, mutation helps to prevent local optimization and to meet the optimization limitation. The scheme of blending genes and mutations is illustrated through an example of parents with ten genes according to Figure 2, where $F_{1}^{\prime }$ and $F_{2}^{\prime }$ stand for the immature offspring.

The genetic algorithm is based on the theory of evolution, where the population consists of the people who persevered and generated offspring [65,66]. The standard that defines the level of adjustment to the environment is the cost/fitness function. In this work, the cost function is the error between the reference result (full-sensor iFEM) and the result of the searched position of sensors through each generation of GA as:

(10) $$f=\sum_{i=1}^{N}\left[\phi_{1}{\left(u_{i}^{j}-u_{i}^{ref}\right)}^{2}+\phi_{2}{\left(v_{i}^{j}-v_{i}^{ref}\right)}^{2}+\phi_{3}{\left(w_{i}^{j}-w_{i}^{ref}\right)}^{2}+\phi_{4}{\left(\theta_{xi}^{j}-\theta_{xi}^{ref}\right)}^{2}+\phi_{5}{\left(\theta_{yi}^{j}-\theta_{yi}^{ref}\right)}^{2}\right]$$

where for a node with the index of $i,u_{i}^{ref},v_{i}^{ref},w_{i}^{ref},\theta_{xi}^{ref}$, and $\theta_{yi}^{ref}$ are the reference solutions and the genetic results at the $j^{th}$ generation for the given node are $u_{i}^{j},v_{i}^{j},w_{i}^{j},\theta_{xi}^{j}$, and $\theta_{yi}^{j}$. Additionally, $\left(\phi_{1},\phi_{2},\phi_{3},\phi_{4},\phi_{5}\right)$ are the coefficients determining the role of each of the kinematic variables in the cost function, and subsequently the optimum positions of the sensors. Through each iteration of the genetic algorithm process, the eligible parents will pass the generation to their offspring, which locate sensors where they make the cost function the minimum.

**Figure 3:**
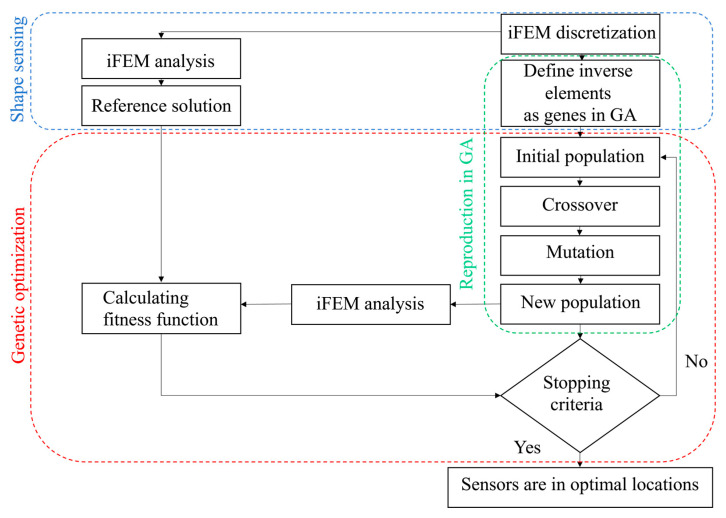
Optimizing the sensor placement using genetic algorithm.

Sensor placement optimization is a restricted problem regarding the number of sensors, so it is required to admit this into the GA problem. The cross-over process may violate the optimization problem restrictions, as shown in Figure 2. This paper assumed that new offspring modify the inherited genes to meet the problems’ condition through the mutation stage. Each individual is correlated with the possible contribution of sensors, and the problem aims to attain the optimum configuration. The reproduction process will be stopped if the best configuration among the population remains constant after a specified number of regenerations. To complete the description, the whole process of the genetic search to find the optimal sensor placement is shown in Figure 3.

## 3. Numerical Examples

In this section, several examples were solved to investigate the accuracy of the iFEM-GA method and determine the optimum arrangement for the considered number of sensors in each case. The results of direct FEM analyses were employed to numerically simulate the experimental strain measures obtained from the in situ strain sensors. The obtained strain data were utilized as the input for the iFEM analysis of each case of study. First, iFEM analysis was performed by allocating sensors in all inverse elements to develop a reference solution for each of the benchmark problems. Then, a cantilever plate with a concentrated load at its free edge was examined to investigate the best number of initial populations for the optimization problem. With the obtained initial population, the number shape sensing of the plate under various boundary conditions was achieved with iFEM-GA having either the whole structure or restricted area of it as the search space. Finally, the practicality of the proposed method was exercised by running the shape-sensing analyses based on the iFEM-GA method for two cases of stiffened plate and curve structure.

### 3.1. Cantilever Plate under Static/Dynamic Transverse Loading

**Figure 4:**
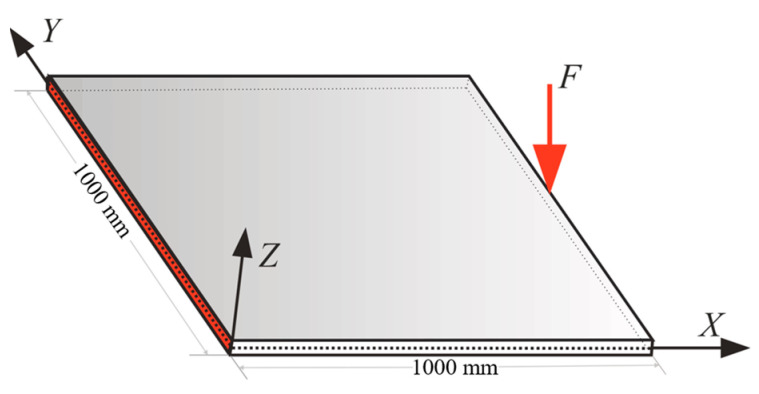
The cantilever plate under transverse loading at the middle of its free edge.

As the first example, the square plate depicted in Figure 4 was examined to see the effect of the location of the sensors and attempted to optimize that using GA. The simulation presented in this section examines the sensor’s best location for the plate with 400 iQS4 elements [30], while only 20% of iQS4 elements (80×2 sensors) carried a sensor. In this case, the left edge of the plate was clamped, and a transverse loading of F=1 N was applied at the middle of the right edge. The thickness of the plate was equal to 3.716 mm with the material properties of E=70 GPa and ν=0.3.

The execution of the genetic algorithm method to detect the best locations for the sensors depends on the number of individuals in the initial population. Therefore, the initial population should be adequate to have sufficient diversity to achieve the optimum result. Some studies have understood the effect of the initial population [67] and their relationship with global optimization; however, this paper attempted to find the proper initial population by studying different cases. Therefore, the convergence of the fitness function was studied for the various numbers of the initial population.

**Figure 5:**
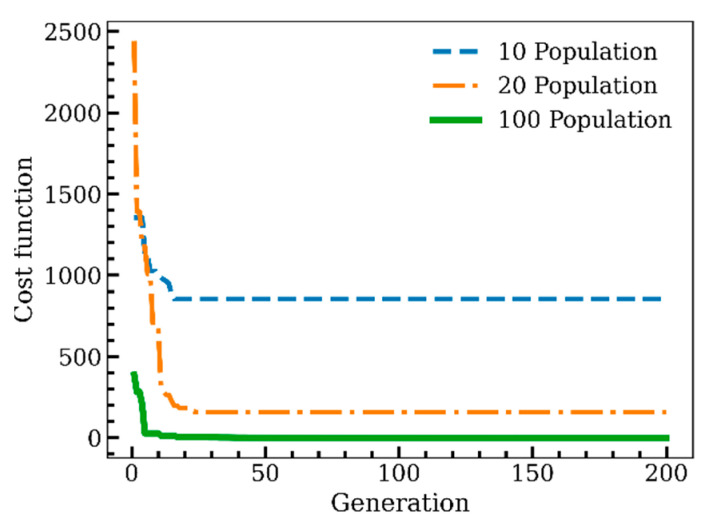
The convergence of the cost function of the optimization problem for different numbers of the initial population.

Figure 5 compares the convergence of the fitness function with the initial 10, 20, and 100 individuals. The fitness function converged to a specific number in all three cases; however, the convergence number, which implies the corresponding cost function for the optimum configuration with the defined initial population, was different. According to Equation (10), the cost function is the square error between the reference solutions and the results obtained through each generation of iFEM-GA analysis. With 10 individuals in the initial population, the cost function converges to a considerable number (cost function = 857), indicating that the optimum configuration of the sensors obtained with genetic algorithms significantly differed from the global optimum. With 20 as the initial population, the error was improved (cost function = 158); nevertheless, the corresponding sensor configuration was not valid. This amount of error is also enormous, indicating that the problem is locally optimized. To dispose of the local optimization, the initial population plays an important role. According to Figure 5, with 100 individuals in the initial population, the cost function converges to a very small number (≈0), showing that the corresponding arrangement of sensors is globally optimized using GA.

The genetic algorithm searches for the most eligible individuals and mainly starts with notable errors, implying that the predecessors do not match the environment and they cannot reproduce; however, after some generations, the error drastically decreases by GA to reach the minimum error, which indicates that the defective genes cannot survive through generations. The randomness of the sufficient initial population can lead to the globally optimal solution of the optimization problem, but it will also make the results of each optimization different, in another words, the optimum configuration of the sensors is not unique [66].

**Figure 6:**
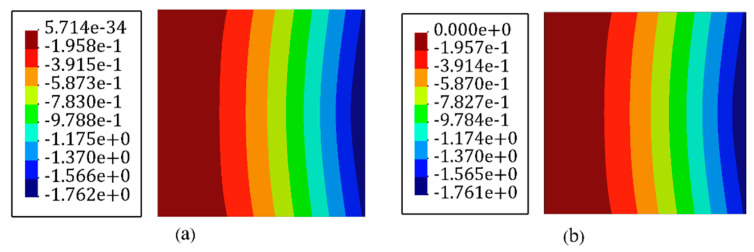
Deflection contours of the plate shown in Figure 4. (**a**) FEM analysis and (**b**) iFEM analysis using the full-sensor placement model.

Based on the procedure established in Figure 3, all of the inverse elements in the iFEM model, primitively, were assumed to be occupied with sensors to achieve the reference solution. In the next step, a high-fidelity FEM analysis was carried out using the commercial software of ABAQUS. The mesh independence study was already performed for finite element analysis, and the most refined mesh consisted of 900 S4R elements that possessed 4500 DOFs. These FEM analyses were utilized to generate the discrete in situ strain measurements that reproduce the strain data obtained from the FBG sensors accommodated at the surfaces of the plate. The accurate reference solution, which is denoted by the ‘ref’ superscript in Equation (10), can be obtained with iFEM with a full-sensor placement model in which each of the iQS4 elements carries a sensor. Since the verification of the new element formulation was the main concern, the accuracy of the reference solution was assessed by comparing the obtained transverse deflection with high-fidelity FEM. Figure 6 depicts the displacement contour plot in the transverse direction using iFEM analysis with the full-sensor placement model and compared it with high-fidelity FEM results. In this comparison, the maximum and minimum deflection locations were in good agreement with the FEM and reference solution. Additionally, the error between FEM and the iFEM maximum deflection was 0.04%, demonstrating that the iFEM reconstruction with the full-sensor placement model agreed with the high-fidelity FEM result and can confidently be considered as the reference solution.

**Figure 7:**
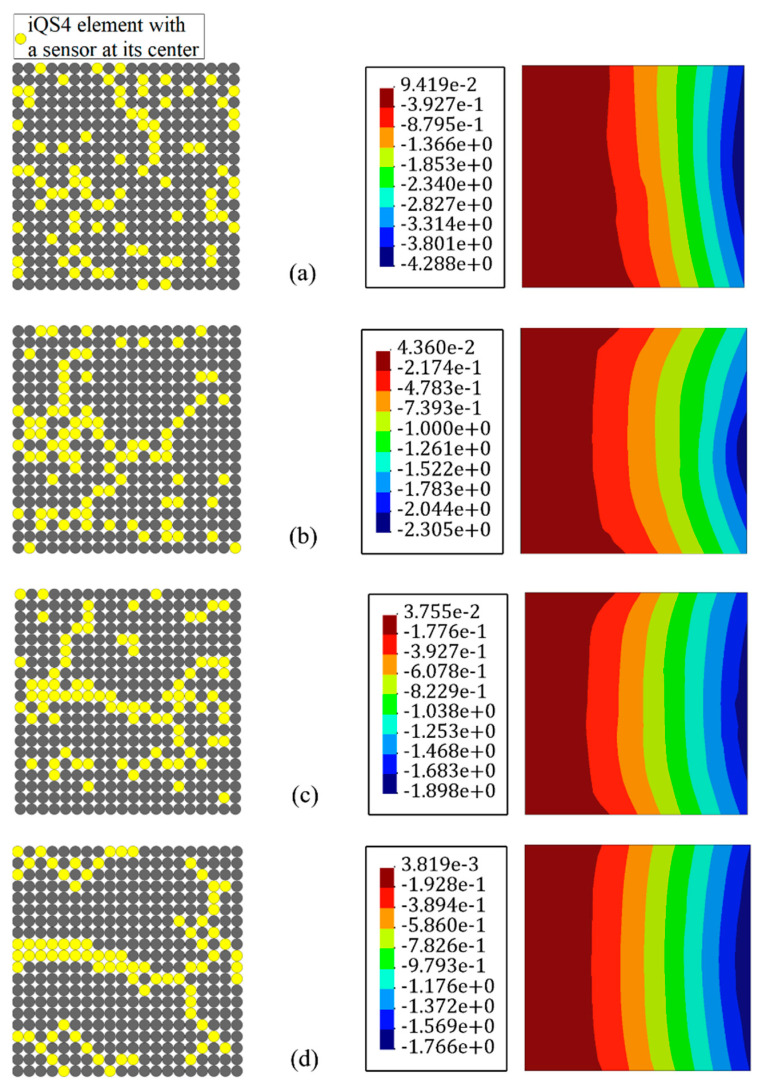
Reconstruction of the transverse displacement with reduced sensors in the iFEM-GA method after (**a**) the first generation, (**b**) 20 generations, (**c**) 50 generations, (**d**) 200 generations.

The required number of sensors for the reference solution is seen to be impractical in the real test environment; hence, this research aimed to reduce the number of sensors while having consistent and accurate inverse finite elements output. In situ section strains are the input of the iFEM, and their values are vital to performing a precise analysis; thus, a GA optimization algorithm was used to detect the optimum locations for the available sensors, which was 20% of all of the iQS4 elements (80×2 sensors in this problem). According to Figure 5, an initial population of 100 is sufficient to minimize the fitness function through 200 generations. However, the problem was also considered after the first, 20, and 50 generations. Figure 7 depicts the sensor configuration through the different generations and the corresponding results of deflection for each arrangement.

After the first generations from the starting point to explore the optimum positions for the available sensors, the obtained results conjugated to the configuration of the sensors according to the Figure 7a, which showed a 143.66% error compared to the reference solution. The error value was considerable and interpreted as insufficient generations in reaching the best state. Through the generations, the population will be created from the most eligible parents, while the offspring of vulnerable parents, which can be considered as defective genes, cannot survive to reproduce in the next generation and fail in natural selection. The error after 20 generations (≈57.96%) compared to that at the beginning showed a drastic drop; this implies that the infirm part of the population is eliminated with a higher rate at the initial stages. According to Figure 7c,d, after 50 and 200 generations, the error reduced to 7.8% and 0.26%, respectively. This small difference with the reference solution implies that the corresponding value of cost function for both generations converges to an insignificant value (Figure 5).

From the point of view of sensor configuration, according to Figure 7a, at the initial generations, the candidate parents for reproduction were mostly separated; however, their offspring became closer to connect and create strings. At the last stage, almost all the sensors were attached and built neuron-like configurations.

**Figure 8:**
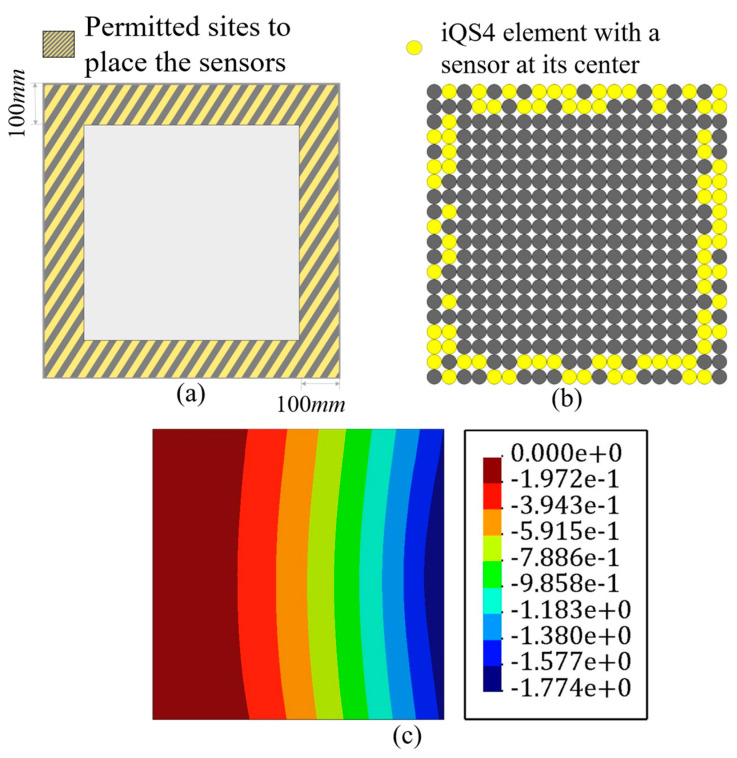
The iFEM-GA results of the plate depicted in Figure 4 with a limited search space. (**a**) The permitted sites to place sensors, (**b**) optimum sensor placement, and (**c**) displacement contours.

There are impediments in deploying sensors, for example, FBG sensors are connected, and their enforced curve is limited. Thus, there is a restriction in the placing of the sensors. Abdollahzadeh et al. [59] suggested some experienced-based feasible sensor configurations for analyzing the structures; however, herein, the goal was to make the results of iFEM-GA more applicable by applying some geometrical constraints to the sensor optimization problem. On this point, as a practical example, Figure 8a depicts the adaptable positions for the available sensors in the plate depicted in Figure 4, and limits the search space of the GA problem to the highlighted part. By confining the search space of the optimization problem, the number of genes for individuals decreased to 25% of the initial number. In other words, the length of the string depicted in Figure 2, shrank by 75%. The GA suggests the sensor distribution shown in Figure 8b as the optimized locations for the plate in Figure 4 with the permitted sites in Figure 8a. According to Figure 8b, GA places sensors evenly in two assigned rows near the edges. The number of sensors in both rows were almost equal, which means that GA attempted various configurations for the sensors to dispose from local optimization. Adjusting all of the sensors in a single line, at first glance, can lead to local optimization; however, since the initial population and generations were properly investigated (Figure 5), GA achieved global optimization with a starting optimization with a sufficiently random gene configuration for the parents at each attempt. With the strain measures collected from the sensors in Figure 8b, the transverse deflection of the plate in Figure 4 was reconstructed using iFEM-GA with a confined search space. According to Figure 8c, even with the geometrical constraint, the error between the obtained results and the reference solution is negligible (≈0.8%), implying that the obtained sensor configuration was the optimum in the designated area.

It is important to note that the initial geometrical constraint imposes a limitation to the search space of the genetic problem. The search space should contain eligible parents to improve their genes in the future generation to produce qualified offspring. Therefore, the initial search space is vital to obtain an admissible result. The minor error with the reference solution indicates that the highlighted area in Figure 8a can constitute eligible parents to achieve the optimum result.

However, running the problem without a spatial constraint in the first stage is highly recommended as its corresponding sensor distribution will demonstrate the best genes to allocate sensors in the optimum offspring’s chromosomes. Hence, if the goal is limiting the search space of the optimization problem, it can be confined to the zones showing the maximum sensor density in the optimization problem without a geometrical constraint.

Moreover, the plate presented in Figure 4 was subjected to the sinusoidal load of $F=-2\cos(\omega_{p}t)-\sin(\omega_{p}t)\;N$, with $\omega_{p}=10.52\;\mathrm{rad}/s$ to assess the practicality of the iFEM-GA in real-time full-field displacement reconstruction of the structures under dynamic loadings. The same discretization and number of sensors in Figure 7d (400 iQS4 elements with 80×2 sensors) were implemented to produce the transverse deflection of the plate under the loading F. The circular frequency of the dynamic loading $\left(\omega_{p}=10.52\;\mathrm{rad}/s\right)$ was reasonably far from the natural frequency of the plate, which was equal to $\omega_{n}=16.45\;\mathrm{rad}/s$ for the first mode, to avoid t he structural resonance and beating phenomenon. The overall structural density was considered as $\rho =2750\;{\mathrm{kg}/m}^{3}$ to apply the mass effect in the problem. The dynamic explicit solver of Abaqus CAE was implemented to calculate the natural frequency of the plate in Figure 4. One of the most advantageous merits of iFEM is providing a pioneering tool for real-time monitoring of the structures. Hence, using the dynamic explicit solver in Abaqus CAE, the sensor data necessary to track structural behavior can be obtained from its high-fidelity FEM counterpart under the dynamic loading of F. To achieve this, the problem is solved at a variety of time intervals so that its history of response can be precisely tracked and exploited as the input for iFEM. The obtained strain measures from the sensors were adjusted according to the configuration in Figure 7d, at each time interval, which were the input to reconstruct the transverse deflection of the structure at the considered instants.

To examine the structural response of the plate and validate the accuracy of the presented method for real-time monitoring, time histories of the displacement predicted by iFEM-GA analysis were compared with the reference solution at two points of $P_{1}\;(x=500\;\mathrm{mm},\;y=1000\;\mathrm{mm})$ and $P_{2}\;(x=500\;\mathrm{mm},\;y=500\;\mathrm{mm})$. The simulation was conducted for 5 s and through this duration, the strain data were collected from the mounted sensors on the surfaces of the plate with the distribution as shown in Figure 7d.

**Figure 9:**
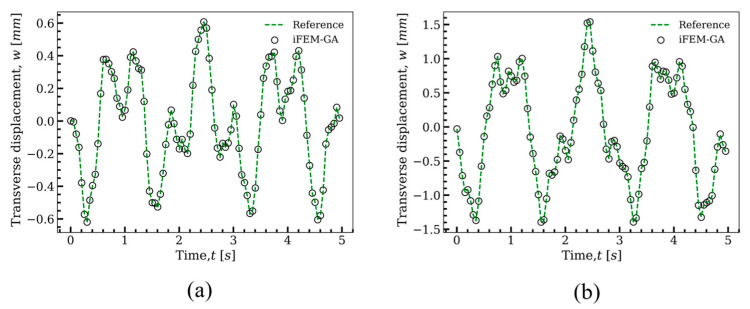
Time history of the transverse displacement of the plate depicted in Figure 4 under the load of $F=-2\cos(\omega_{p}t)-\sin(\omega_{p}t)\;N$, with $\omega_{p}=10.52\;\mathrm{rad}/s$. A comparison of the reference solution and iFEM-GA analysis at position (**a**) $P_{1}\;(x=500\;\mathrm{mm},y=1000\;\mathrm{mm})$, (**b**) $P_{2}\;(x=500\;\mathrm{mm},y=500\;\mathrm{mm})$.

The time–domain variations of the transverse displacement, w, calculated at $p_{1}$ and $p_{2}$ are presented in Figure 9a,b, respectively. In these figures, the duration of the analysis was divided into 100 time intervals and at each time increment, the transverse displacements of $p_{1}$ and $p_{2}$ obtained with iFEM-GA, were clearly compared with the continuous results of the reference solution. Each empty mark denotes the response of the point at the specified instant. Significantly, the results of the reference solution and the proposed method for the transverse deflection in the considered locations were almost indistinguishable from each other. The iFEM-GA model predicted highly accurate transverse displacements at these points, utilizing sensors only in 20% of the iQS4 elements, which fairly agreed with reference solution.

**Figure 10:**
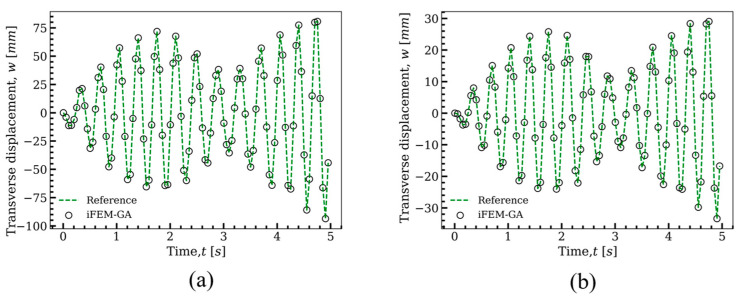
Time history of the transverse displacement of the plate depicted in Figure 4 under the load of $F=5\sin(\omega_{p}t)$ with $\omega_{p}=18.83\;\mathrm{rad}/s$. A comparison reference solution and iFEM-GA analysis at position (**a**) $P_{1}\;(x=500\;\mathrm{mm},y=1000\;\mathrm{mm})$, (**b**) $P_{2}\;(x=500\;\mathrm{mm},y=500\;\mathrm{mm})$.

To further investigate the practicality of the obtained sensor configuration in Figure 7d to capture the dynamic response of the plate in the Figure 4 under the applied load with various frequencies and amplitudes, the concentrated load changed to $F=5\sin(\omega_{p}t)$ with the loading frequency of $\omega_{p}=18.83\;\mathrm{rad}/s$. This loading frequency is fairly close to the natural frequency of the plate $\left(\omega_{n}=16.45\;\mathrm{rad}/s\right)$ to impose the beating phenomenon to the structure. According to this phenomenon, the maxima and minima of the response were no longer constant and changed over time. When the natural and loading frequencies were nearly 180° out of phase, the maxima of one wave cancelled the minima of the other, whereas when they were nearly in phase, their maxima were summed up, raising the perceived volume. Figure 10 depicts the displacement response of the plate at two different points of $P_{1}\;(x=500\;\mathrm{mm},\;y=1000\;\mathrm{mm})$ and $P_{2}\;(x=500\;\mathrm{mm},\;y=500\;\mathrm{mm})$ under the applied load. According to this figure, the results obtained with the suggested sensor configuration in Figure 7d through the iFEM-GA procedure were almost indistinguishable with that in the reference solution. Therefore, this proves the remarkable performance of the proposed method to track the structural response under loadings with various amplitudes and frequencies utilizing an identical sensor distribution pattern. Overall, these results validate the accuracy of the iFEM-GA method for usage in the real-time displacement monitoring of structures with a fewer number of sensors.

### 3.2. Cantilever Plate under Concentrated Loading at Its Corner

**Figure 11:**
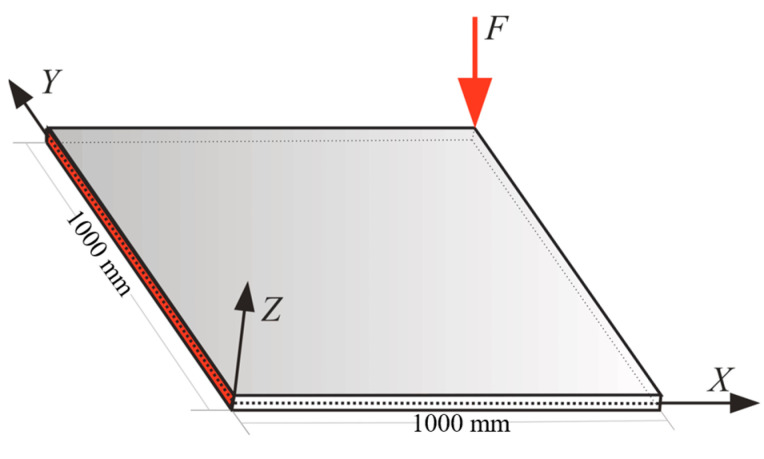
The cantilever plate under the concentrated force at its corner.

To examine the performance of the genetic algorithm in detecting the optimum positions for the available sensors to maintain the accuracy of the results of iFEM with a reduced number of sensors, the square plate in Figure 4 was further investigated under different boundary conditions and loading types. Figure 11 describes a cantilever plate (fixed at the shaded edge) subjected to a transverse loading, F=1 N, at the top right corner. 

**Figure 12:**
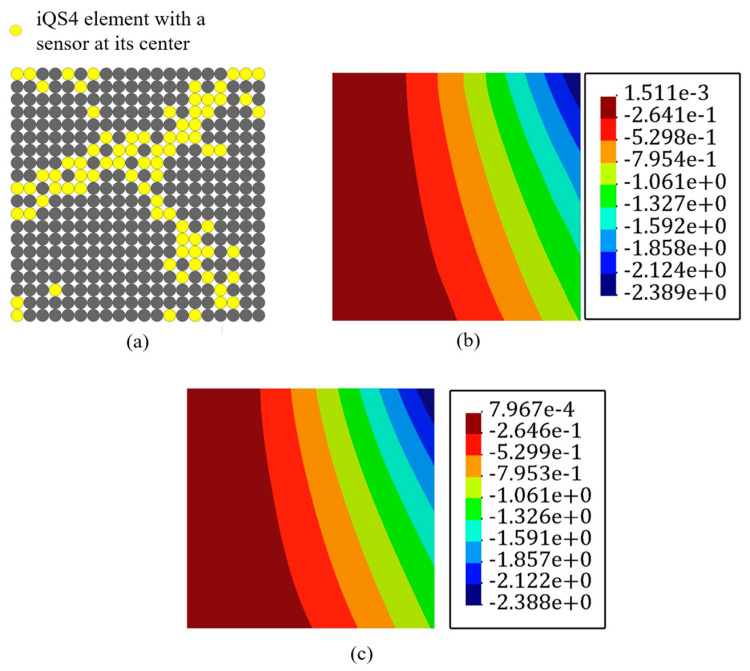
(**a**) Optimum sensor configuration obtained for the plate shown in Figure 11. (**b**) Transverse displacement contour obtained with iFEM-GA, (**c**) reference solution.

**Figure 13:**
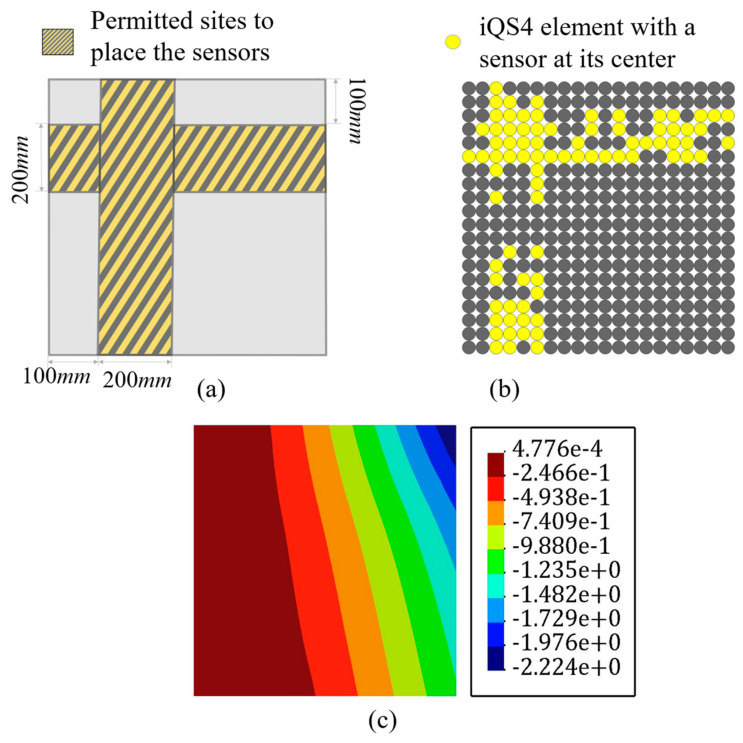
The iFEM-GA results of the plate depicted in Figure 10 with limited search space. (**a**) The permitted sites to place sensors, (**b**) optimum sensor placement, and (**c**) displacement contours.

**Figure 14:**
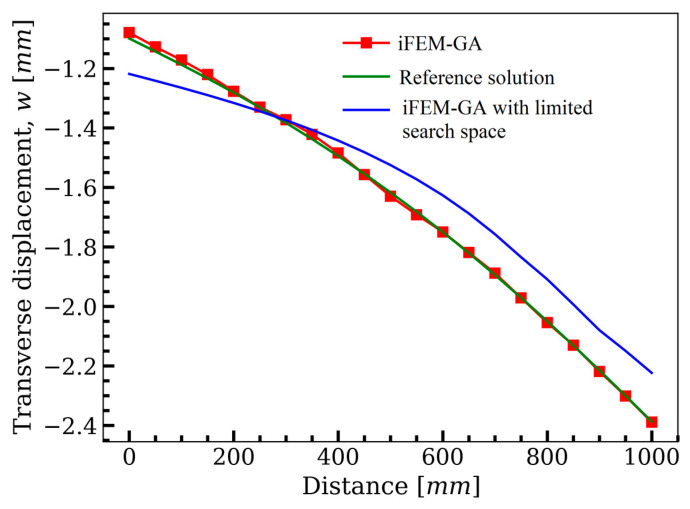
The comparison of the deflection of the plate in Figure 10, path obtained with iFEM-GA analysis, iFEM-GA analysis with limited search space, and reference solution through the defined path.

Following the results obtained in Figure 5, the iFEM-GA algorithm was initiated with 100 individuals as its initial population and iterated up to the maximum generation of 200. Note that the first constraint of the optimization problem imposes a definite number of sensors (20% of the inverse elements) as the volume fraction. Therefore, within the search domain of the plate, the genetic algorithm suggests the optimum locations for the available sensors, as shown in Figure 12a. Similar to that shown in Figure 7d, sensors become closer to connect and build a neuron-like string. Although this string propagates from the fixed edge of the plate, unlike the sensor configuration in Figure 7d, it does not continue in a straight path to be branched toward the upper and lower surface. The created string in Figure 12a turned to the corner, where the maximum displacement occurred. Another secondary branch moved toward the lower corner in the symmetric position of the loading point. The sensor depicted a higher concentration in the vicinity of the maximum displacement in the suggested optimum configuration obtained with GA. Figure 12b,c depicts the transverse deflection obtained using iFEM-GA analysis and the reference solution, respectively. According to these figures, the discrepancy between the maximum deflection obtained with iFEM-GA analysis and the reference solution was almost negligible (less that 1%). Regarding the transverse displacement contours, both iFEM-GA and the reference solution presented an almost similar distribution. However, in Figure 13, the number of sensors was the only restraint applied to the optimization problem; thus, GA explored the optimum locations all over the domain. Nevertheless, in a practical example, Figure 13a limited the search space to the highlighted parts. In this case, the suggested sensor configuration concerning the imposed geometrical restrictions in the search domain and the corresponding reconstructed deflection are depicted in Figure 13b,c, respectively. According to Figure 13b, the sensors tended to show a higher concentration near the free edges of the plate, where they experienced higher amounts of deflection, rather than creating strings in the inner parts of the permitted site. Figure 13c shows consistency in the maximum values of w in the iFEM-GA analysis with a confined search space and the reference solution in Figure 12c as well as its corresponding contour. The equivalent error between the maximum values of transverse deflection was equal to 6%. However, this agreement was not valid over the area of the plate. To better comprehend the deviation of the results acquired by GA optimization from the reference solution for both analyses with the whole domain and constrained area as the search space, the transverse deflection was plotted through the path that connects the middle of the side edges according to Figure 14. The GA results with the entire domain as the search space agreed with the reference solution very well; however, with the geometrical restriction, the transverse deflection had considerable deviation from the reference solution, which can be disregarded concerning the goal of the simulation.

### 3.3. Partially Clamped Plate under Concentrated Loading at Its Center

**Figure 15:**
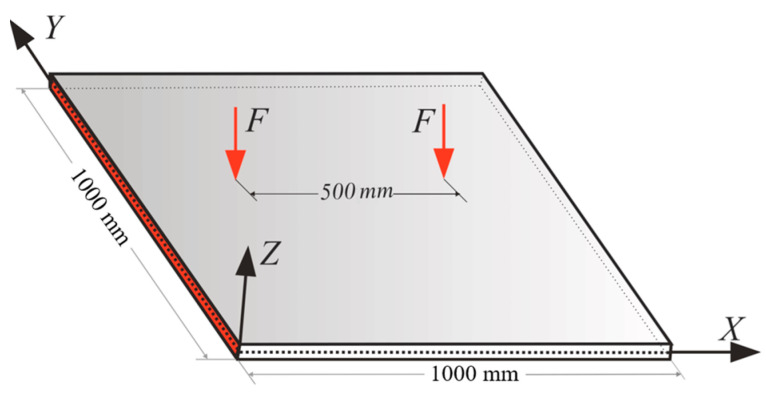
The partially clamped plate under two concentrated forces.

**Figure 16:**
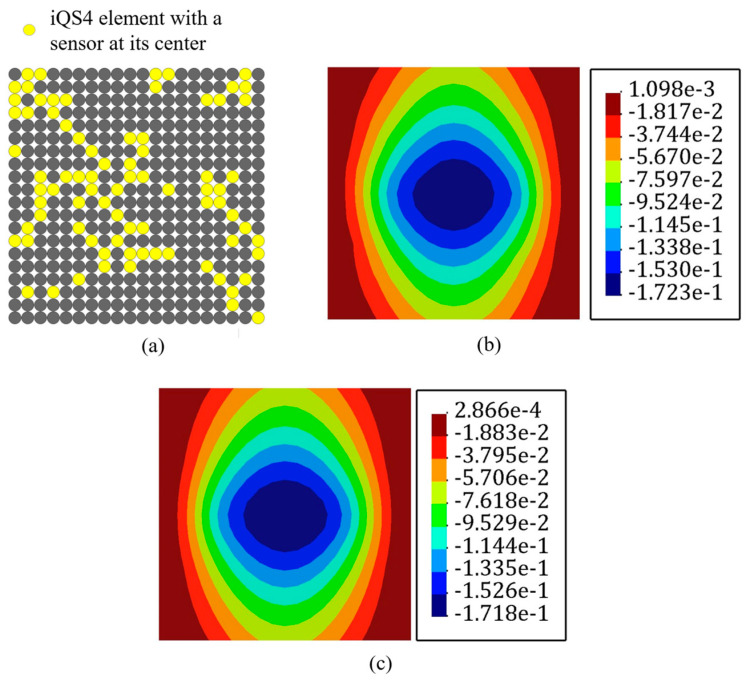
(**a**) Optimum sensor configuration obtained for the plate shown in Figure 14. (**b**) Transverse displacement contour obtained with iFEM-GA, (**c**) reference solution.

**Figure 17:**
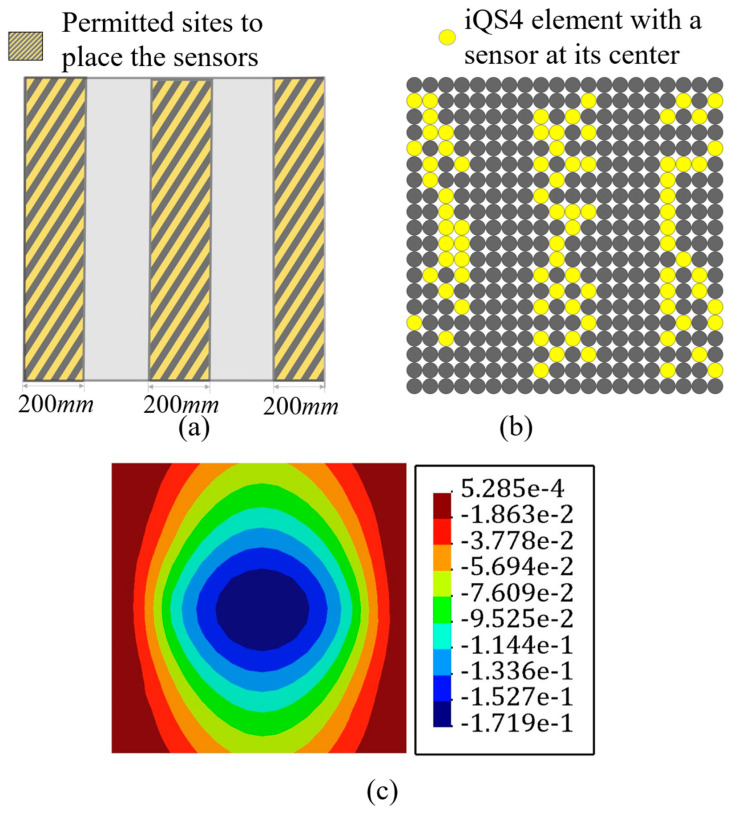
The iFEM-GA results of the plate depicted in Figure 15 with limited search space. (**a**) The permitted sites to place sensors, (**b**) optimum sensor placement, and (**c**) the displacement contours.

**Figure 18:**
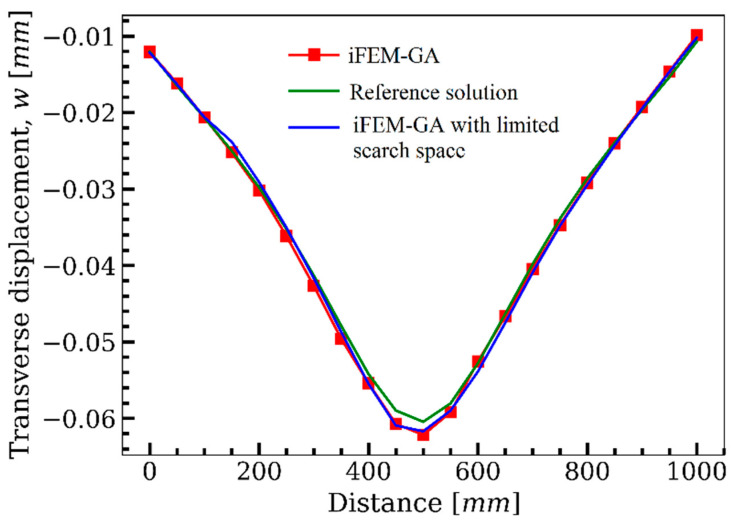
The comparison of the deflection of the plate in Figure 15 obtained with iFEM-GA analysis, iFEM-GA analysis with limited search space, and the reference solution through a defined path.

Figure 15 represents the square plate depicted in Figure 4, which was clamped at its left and right edges. The iFEM-GA method attempts to reconstruct the real-time full-field displacement of this plate under two concentrated forces, F=5 N, applied at an equal distance from both side edges. To achieve the best accuracy of the results with iFEM-GA analysis, where only 20% of thee iQS4 elements carry sensors, the sensor placement model in Figure 16a was suggested by genetic optimization. In this configuration, the sensors create strings initiated from the clamped edges to join in the center of the plate. Figure 16b depicts the reconstruction of the transverse deflection of the plate utilizing the strain data collected from the sensors distributed according to Figure 16a. While the reference solution depicts a smoother transverse displacement contour in comparison to that iFEM-GA, there was a small inaccuracy between the obtained maximum deflection with either method (≈1%). Although the obtained result in Figure 16 using the proposed method showed good agreement with the reference solution, organizing the sensors in the offered composition in Figure 16a is challenging. Therefore, considering the obtained configuration using the reduced iFEM-GA methodology, the constraint in Figure 17a was applied to the search domain to achieve a more practical result. Figure 17b suggests the optimum locations for the sensors in the constrained area, and the corresponding real-time deflection reconstructed using the data obtained from these sensors is depicted in Figure 17c. According to the sensor configuration demonstrated in Figure 17a, the sensors tended to be located in the regions closer to the center of the plate, which showed higher displacement values. Accordingly, in the three separated areas, a higher portion of iQS4 elements in the inner columns was occupied by sensors. The maximum value of w obtained with iFEM-GA analysis with the confined search domain in Figure 17c showed a negligible error with the iFEM-GA results and reference solution in Figure 16b,c, respectively. Moreover, to better compare the results obtained by full-sensor iFEM, reduced iFEM-GA, and constrained iFEM-GA, the deviation of the results using iFEM-GA and iFEM-GA with limited search space from the reference solution is depicted in Figure 18. According to these comparisons, the iFEM-GA models, with both the whole and confined search spaces, produced almost the same transient trend with a small deviation from the reference solution at the middle of the plate. Near the edges, the obtained results were almost indistinguishable.

**Figure 19:**
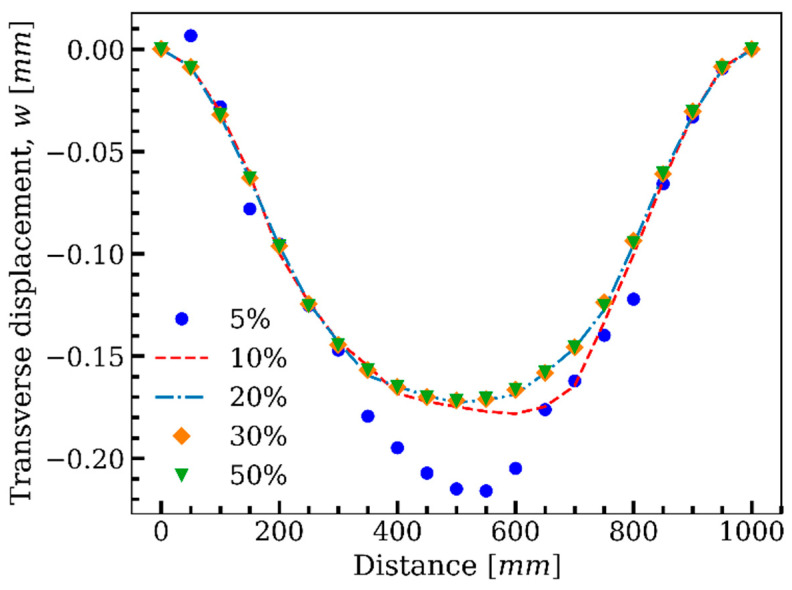
The effect of the ratio of the available sensors to the number of inverse elements on the accuracy of the results.

Moreover, the effect of the ratio of the iQS4 elements with sensors to the number of all inverse elements in the plate shown in Figure 15, $\nu_{f}$, in the accuracy of the results was further investigated. Herein, the plate was analyzed with five sensor volume ratios of $\nu_{f}=0.05,0.1,0.2$ and 0.3, and compared with that of the reference solution. Subsequently, GA sought the optimum positions for the available sensors in the defined search space according to the geometry of the plate in Figure 15. Then, the obtained results with the considered sensor volume ratios were compared with the reference solution. Additionally, Figure 19 depicts the transverse displacement of the middle of the plate through the length with different sensor volume ratios.

The transverse deflection was reconstructed using the data gained by the sensors residing in the optimum elemental locations determined by GA for each volume fraction. With sensors in only 5% of the inverse elements, the results deviated considerably from the reference solution. By increasing the number of sensors to 10% of the inverse elements, the achieved results became closer to the reference solution; however, there was still divergence from the ideal case. For the case of 20% of inverse elements with sensors and further, GA located the sensors in the locations where the discrepancy from the reference solution was negligible, which means that for the plate shown in Figure 14, GA could construct the displacement by 20% of sensors $\left(\nu_{f}=0.2\right)$, with the same accuracy of the full-sensor condition.

### 3.4. Fully Clamped Plate under Uniform Pressure

**Figure 20:**
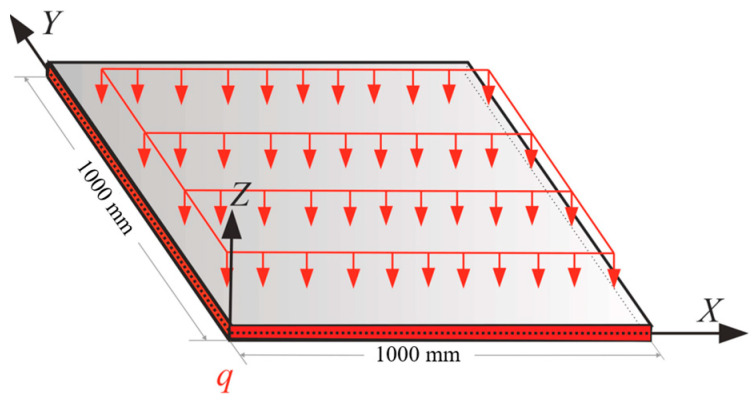
The clamped plate under a distributed load.

**Figure 21:**
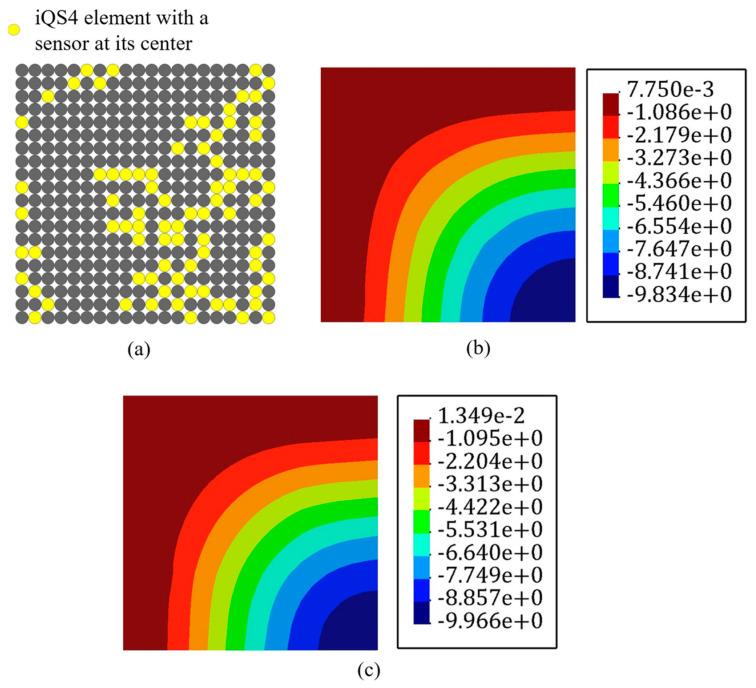
(**a**) Optimum sensor configuration obtained for the plate shown in Figure 19. (**b**) Transverse displacement contour obtained with iFEM-GA, (**c**) reference solution.

**Figure 22:**
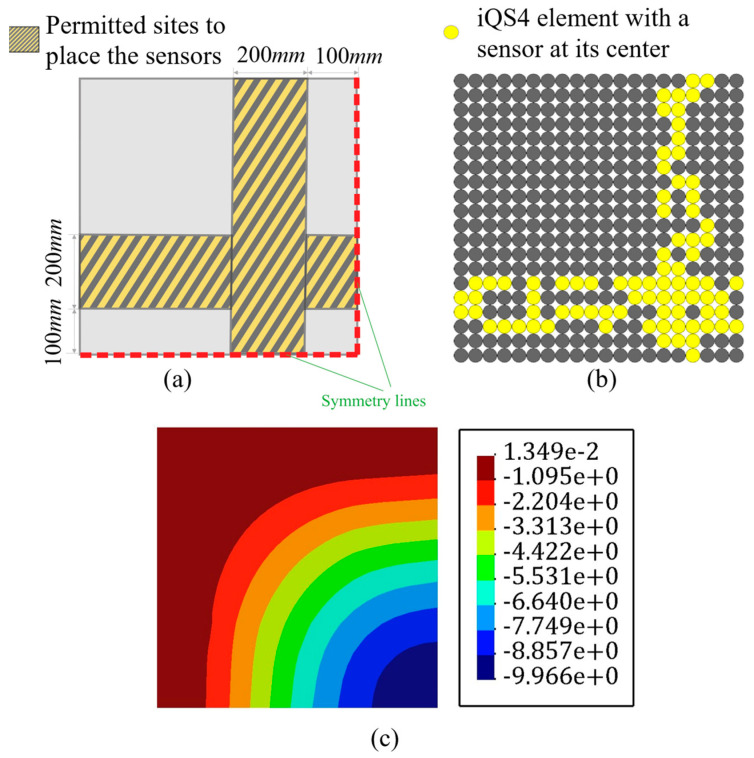
The iFEM-GA results of the plate depicted in Figure 19 with limited search space. (**a**) The permitted sites to place sensors, (**b**) optimum sensor placement, and (**c**) displacement contours.

**Figure 23:**
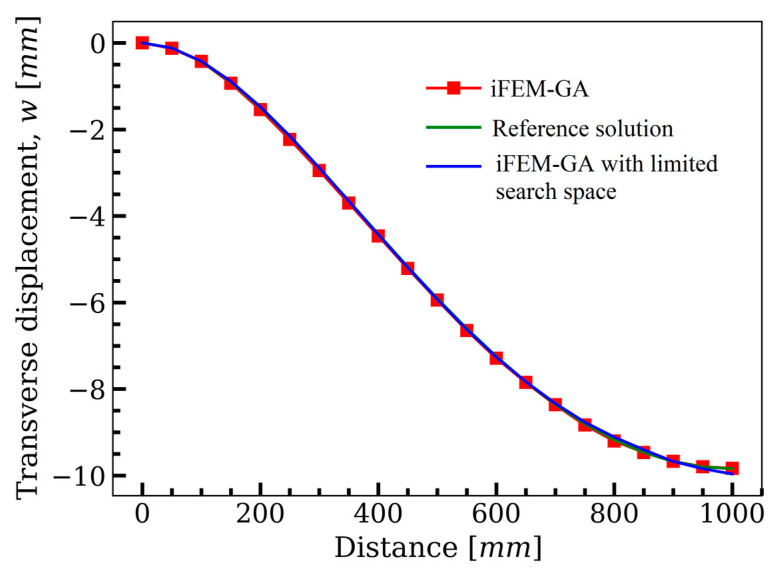
The comparison of the deflection of the plate in Figure 20 obtained with iFEM-GA analysis, iFEM-GA analysis with limited search space, and the reference solution through a defined path.

In this example, the aim was to study the case in which all oof the discretizing nodes carried the applied load. In other words, the plate in Figure 4 was studied under a distributed load of $q=0.1KN/m^{2}$. Figure 20 depicts the loading condition of this case, where all the plate edges were fully clamped. Due to the symmetric boundary condition, only one-fourth of the geometry of the plate shown in Figure 20 was simulated to reduce the computational costs. In this example, 20% of iQS4 elements was occupied with sensors. Figure 21a expresses the optimum locations for these sensors obtained using genetic optimization. According to this configuration, the main portion of the available sensors was concentrated at the center of the plate, which demonstrates the maximum value of the transverse deflection. Figure 21b depicts the reconstructed displacement and a comparison with the reference solution shown in Figure 21c. In this case, the discrepancy between the iFEM-GA results and the reference solution was negligible; however, the suggested sensor position by GA (with the whole domain as the search space) had shortcomings regarding the limitation of the utilized sensors. Therefore, to make the proposed method practical, the constraint demonstrated in Figure 22a is suggested to apply to the geometry. Because of the features of the many sensors, allocating them in series is preferable rather than having a scatter distribution of the sensors. Therefore, the permitted sites in Figure 22a are suggested as the search space for the optimization problem. Figure 22b shows the obtained optimum locations for the sensors in the allowed region. The number and variation in the eligible parents were reduced as a result of confining the search space. However, the maximum transverse displacement predicted using the iFEM-GA method after diminishing the search space had a 1.5% deviation from the reference solution, as can be seen from Figure 22c. Additionally, as demonstrated in Figure 23, the deflection of the plate obtained using iFEM-GA with both the complete and limited search space, through the horizontal symmetric line, was almost identical. Overall, these comparisons validate and demonstrate the superior predictive capabilities of iFEM-GA analysis in utilizing a fewer number of sensors in comparison with the reference solution.

### 3.5. Stiffened Plate and Curved Shell Structures

**Figure 24:**
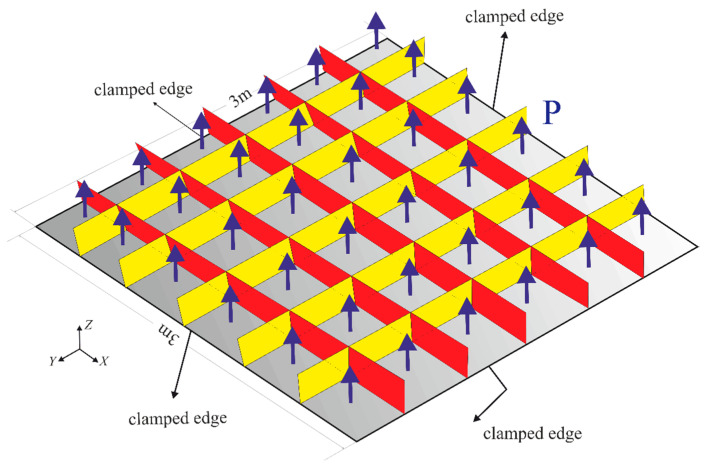
Clamped stiffened plate geometry under distributed load.

To further investigate the practical application of iFEM-GA to the real-time displacement monitoring of complex structures, two samples of a stiffened plate and a curved shell were studied herein. In fact, the structure of the studied stiffened plate can be representative of a floating marine structure, therefore, the following iFEM-GA analysis can be beneficial for the real-time SHM capabilities of marine structural components utilizing fewer sensors. The stiffened plate consists of a plate as the immediate member and beams as the secondary member. These are generally used for weight saving and increasing the stability in various structures. Herein, a stiffened square plate with the material property of E=210 GPa and ν=0.3 was determined to be solved based on the proposed method. The square plate with a length of 3 m and uniform thickness of 15 mm was clamped from all edges, as depicted in Figure 24.

**Figure 25:**
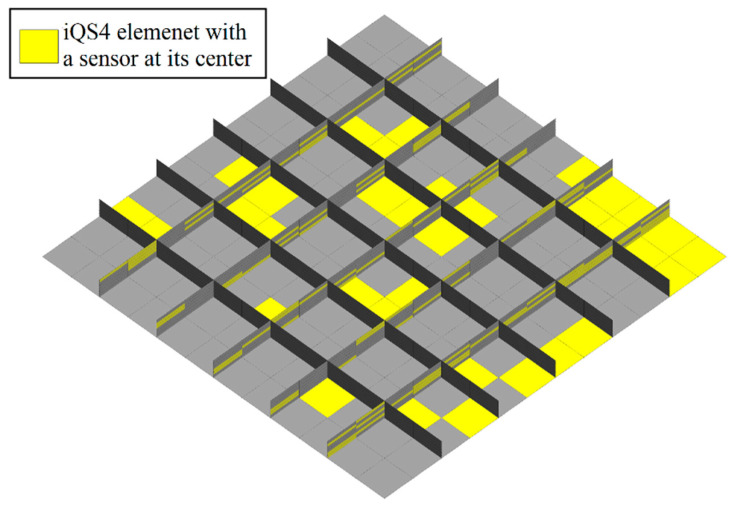
Optimized sensor distribution for the stiffened plate obtained with the iFEM-GA method.

A static uniform transverse pressure of P=40 KPa is subjected to the bottom surface of the plate. Moreover, mesh independent FEM results have been conducted to extract the strain values of in-situ strain gauges. In the FEM study using the commercial software of Abaqus, 4284 square and uniformly distributed S4 (four-node shell) elements are implemented to solve the problem. Besides, 864 iQS4 elements carry sensors at their top and bottom surface to reconstruct the real-time full-field transverse displacement of the stiffened plate with full-sensor iFEM methodology. In the following, the number of sensor elements was reduced to 30% of all iQS4 elements (259×2 sensors). The GA optimization was carried out with 100 individuals as the initial population, which suggests Figure 25 as the optimum locations for the available sensors.

**Figure 26:**
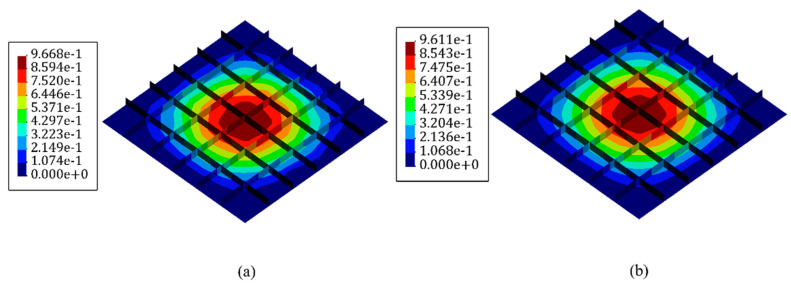
The transverse displacement contour of the stiffened plate with (**a**) iFEM-GA analysis and (**b**) the reference model.

Generally, stiffeners run constantly through the supporting frame and are in charge of strengthening that and experiencing the maximum stress values and consequence strains. Thus, the value of extracted strains from the elements in these regions plays a crucial role in the accuracy of the obtained results. Therefore, this observation affirms the concentration of the available sensors on stiffeners. In Figure 26, the variation in the displacement contours for both iFEM-GA and the reference models were almost indiscernible from each other. The percentage error between the maximum values of the displacement was less than 0.5%. This comparison indicates that the iFEM-GA formulation enabled a highly accurate reconstruction of the deformed shape of the structure with a reduced number of strain measurements.

**Figure 27:**
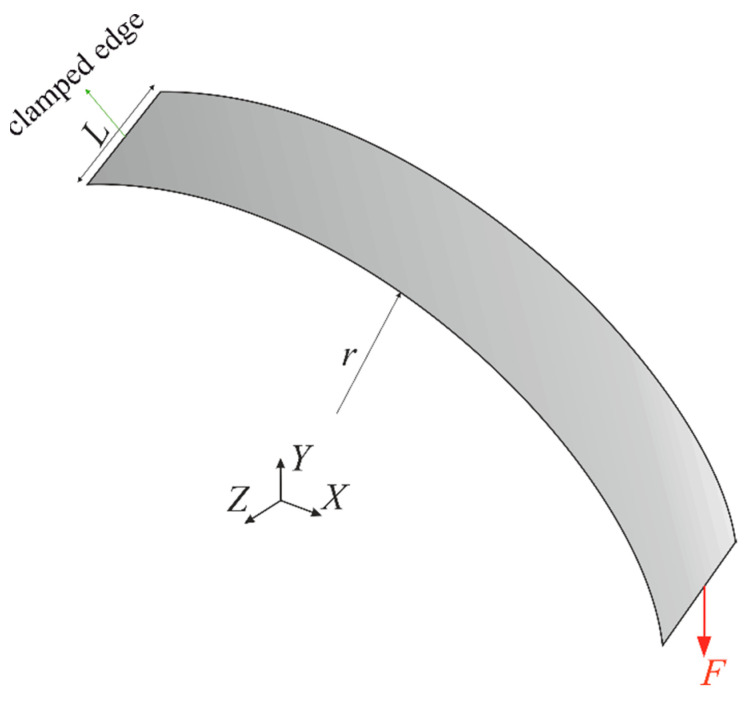
Geometry of the thin-walled cylinder under a concentrated force.

In the previous examples, the robustness of the proposed method was assessed by way of flat-shell and stiffened plate problems; however, the advantage of curved shell plates in many practical engineering applications is highlighted. Therefore, a thin-walled cylinder with a radius of r=100 mm, a length of L=20 mm, and a uniform thickness of 2h=1.5 mm was examined to indicate the reliability of the iFEM-GA method for modeling realistic shell structures. The curve plate with E=210 GPa and ν=0.3 was subjected to a concentrated load equal to F=100 N at the middle of the front edge as depicted in Figure 27. A global Cartesian coordinate system of (X,Y,Z) with the origin of (0,0,0) was located at the center of the right-hand-side circle, and positive directions of the coordinate axes are clearly shown in Figure 27.

**Figure 28:**
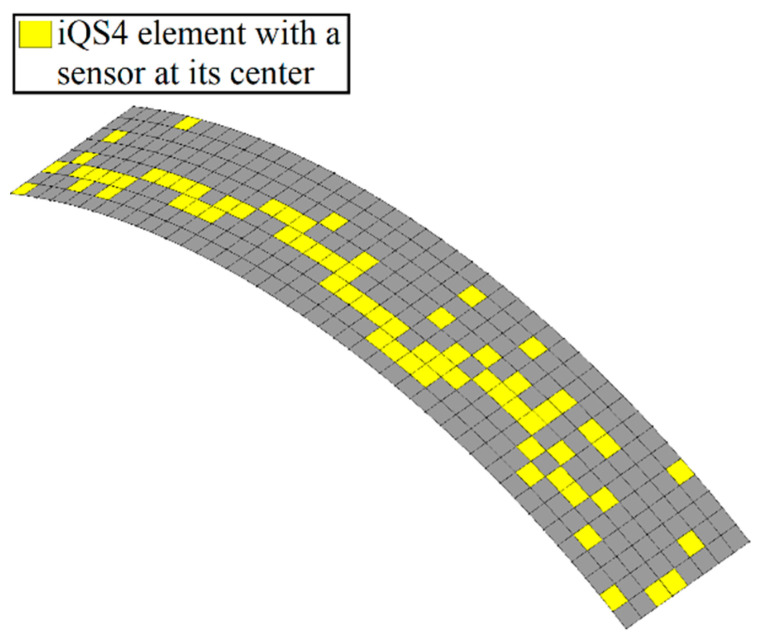
Optimized sensor distribution for the thin-walled cylinder obtained with the iFEM-GA method.

In this structure, 336 iQS4 elements were taken to discretize the geometry. However, only 20% of the inverse elements carried sensors at their top and bottom surfaces (68×2 available sensors). The proposed method attempts to conduct real-time shape sensing of the curve plate by placing the available sensors in the optimum locations suggested in Figure 28.

As discussed in Figure 5, the number of the initial population plays a vital role in the accuracy and consistency of the results as it provides sufficient diversity to proceed with the most eligible parents. An initial population with 100 individuals ensured global optimization and mature convergence after 200 generations in the studied cases.

**Figure 29:**
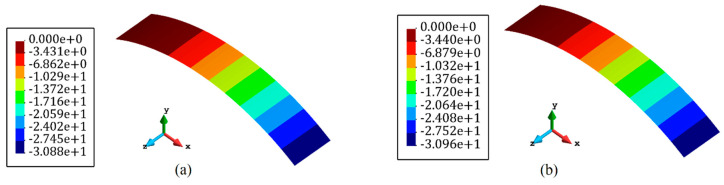
The transverse displacement contour of the curve plate with (**a**) iFEM-GA analysis and (**b**) reference model.

According to the contour plots in Figure 29, the discrepancy between the results obtained with iFEM-GA analysis and the reference solution was less than 1%. These results indicate the superior accuracy of the proposed method in reconstructing the deformation of the thin-walled cylindrical shell-plate with a reduced number of sensors. Therefore, the sparse distribution of sensors could perform very accurate shape sensing of a thin-walled cylinder, which was as accurate as those predicted by the reference model. The deformed shape produced by the GA-suggested configuration in Figure 29a was almost the same as those with the reference shape in Figure 29b, demonstrating the superior practical capability of the iFEM-GA framework. Overall, the potential and versatile applicability of the iFEM-GA methodology was demonstrated for the shape sensing of complex structures subjected to distributed and concentrated loads.

## 4. Concluding Remarks

A combination of the inverse finite element and the genetic algorithm was employed to reconstruct the real-time displacement field of the structures with a reduced number of sensors. GA searched for the optimum locations for the available sensors to minimize the deviation of the iFEM results with a reduced number of sensors from the reference solution (iFEM with sensor in all inverse elements) through the iFEM-GA method. GA employed binary numbers to designate the sensors in iQS4 elements to achieve the optimum configurations for the available sensors, recognizing each element as the genes. The genes represented by one indicate the existence of a sensor in the corresponding element, and the sensors were eliminated from the genes labeled with zero. All genes together are considered as an individual, and a pair of these individuals, known as parents, mix their genes to produce eligible offspring that adjust better to the environment and lead the sensor configuration to minimize the cost function. Genetic optimization has been linked to iFEM by using the constant values used in the iFEM formulation to adjust the coherence between the experimental and analytical strain values. The cost function in the current study was taken as the error between the results of each solving step of GA and the reference solution.

Genetic algorithm initiates the optimization problem with a defined number of individuals known as the initial population, wherein this research, the proper size of the initial population was determined to avoid unmatured convergence. To examine the accuracy of the concept, a plate with different boundary conditions and static/dynamic loading types with only 20% of the inverse elements containing sensors were considered with and without geometrical constraints. The results with a reduced number of sensors showed good consistency with the reference solution in all of the case studies. Furthermore, the effect of the number of sensors on the accuracy of the results was also examined. Utilizing the strain data obtained from 30% of sensors and above, the iFEM-GA results showed almost identical results with the reference solution. Two examples of stiffened plates and curved structures were considered to examine the performance of the method in shape sensing the complicated structures. As a result of optimum sensor locations, the real-time full-field displacement reconstruction with the remaining sensors manifested a consistency with the reference solution with a minor error. Hence, the GA-iFEM methodology is a promising framework for performing accurate shape sensing, providing a viable technology for the SHM of future structures.

## Figures and Tables

**Table 1:** Mechanical properties of the orthotropic and isotropic materials.

Individual	$\mathrm{Gene}\;\mathrm{Value}\;\mathrm{for}\;\mathrm{Each}\;\mathrm{Element},\;\tau_{e}$				Sum of Gene Values, $\sum_{e=1}^{nel}\tau_{e}$
	e=1	e=2	…	e=nel	
1	1	0	…	1	$n_{total}$
2	0	0	…	1	$n_{total}$
…	…	…	…	…	…
λ	1	1	…	1	$n_{total}$

## References

[1] Balageas D., Fritzen C.P., Güemes A. (2010). Structural Health Monitoring.

[2] Wild G., Pollock L., Abdelwahab A.K., Murray J. (2021). The Need for Aerospace Structural Health Monitoring: A review of aircraft fatigue accidents. Int. J. Progn. Health Manag..

[3] Cao Y., Tan W., Wu Z. (2018). Aircraft icing: An ongoing threat to aviation safety. Aerosp. Sci. Technol..

[4] Mir-Haidari S.E., Behdinan K. (2021). Application and implementation of the bond graph methodology on the structural damage detection and monitoring of aeroengines. Aerosp. Sci. Technol..

[5] Wu B., Liu B.J., Zheng J., Wang T., Chen R.G., Chen X.L., Zhang K.S., Zou Z.Q. (2018). Strain-based health monitoring and remaining life prediction of large caliber gun barrel. Measurement.

[6] Saade M., Mustapha S. (2020). Assessment of the structural conditions in steel pipeline under various operational conditions—A machine learning approach. Measurement.

[7] Fang F., Qiu L., Yuan S., Ren Y. (2019). Dynamic probability modeling-based aircraft structural health monitoring framework under time-varying conditions: Validation in an in-flight test simulated on ground. Aerosp. Sci. Technol..

[8] Gethin W.R., Meng X.L., Alan H.D. (2004). Integrating a global positioning system and accelerometers to monitor the deflection of bridges. J. Surv. Eng..

[9] Zhang S., Liu B., He J. (2019). Pipeline deformation monitoring using distributed fiber optical sensor. Measurement.

[10] Brownjohn J.M., De Stefano A., Xu Y.L., Wenzel H., Aktan A.E. (2011). Vibration-based monitoring of civil infrastructure: Challenges and successes. J. Civ. Struct. Health Monit..

[11] Rehman S.K., Ibrahim Z., Memon S.A., Jameel M. (2016). Nondestructive test methods for concrete bridges: A review. Constr. Build. Mater..

[12] Farrar C.R., Doebling S.W., Nix D.A. (2001). Vibration—Based structural damage identification. Philos. Trans. R. Soc. Lond. Ser. A Math. Phys. Eng. Sci..

[13] Bengherbia B., Zmirli M.O., Toubal A., Guessoum A. (2017). FPGA-based wireless sensor nodes for vibration monitoring system and fault diagnosis. Measurement.

[14] Kim H.J., Jang B.S., Du Kim J. (2020). Fatigue-damage prediction for ship and offshore structures under wide-banded non-Gaussian random loadings part I: Approximation of cycle distribution in wide-banded gaussian random processes. Appl. Ocean Res..

[15] Tabrizi I.E., Kefal A., Zanjani J.S., Yildiz M. (2022). Damage growth and failure detection in hybrid fiber composites using experimental in-situ optical strain measurements and smoothing element analysis. Int. J. Damage Mech..

[16] Kuang K.S., Quek S.T., Koh C.G., Cantwell W.J., Scully P.J. (2009). Plastic optical fibre sensors for structural health monitoring: A review of recent progress. J. Sens..

[17] Lynch J.P., Loh K.J. (2006). A summary review of wireless sensors and sensor networks for structural health monitoring. Shock Vib. Dig..

[18] Mascarenas D.L., Todd M.D., Park G., Farrar C.R. (2007). Development of an impedance-based wireless sensor node for structural health monitoring. Smart Mater. Struct..

[19] Gherlone M., Cerracchio P., Mattone M. (2018). Shape sensing methods: Review and experimental comparison on a wing-shaped plate. Prog. Aerosp. Sci..

[20] Abdollahzadeh M.A., Kefal A., Yildiz M. (2020). A comparative and review study on shape and stress sensing of flat/curved shell geometries using C_0_-continuous family of iFEM elements. Sensors.

[21] Esposito M., Gherlone M. (2021). Material and strain sensing uncertainties quantification for the shape sensing of a composite wing box. Mech. Syst. Signal Process..

[22] Esposito M., Gherlone M., Marzocca P. (2021). External loads identification and shape sensing on an aluminum wing box: An integrated approach. Aerosp. Sci. Technol..

[23] Wang X., Si C., Wang Z., Li Y. (2021). Displacement field reconstruction of structures under thermal and mechanical loading environment. Aerosp. Sci. Technol..

[24] Tessler A., Spangler J.L. (2005). A least-squares variational method for full-field reconstruction of elastic deformations in shear-deformable plates and shells. Comput. Methods Appl. Mech. Eng..

[25] Wang J., Ren L., You R., Jiang T., Jia Z., Wang G.X. (2021). Experimental study of pipeline deformation monitoring using the inverse finite element method based on the iBeam3 element. Measurement.

[26] Li M., Kefal A., Cerik B.C., Oterkus E. (2020). Dent damage identification in stiffened cylindrical structures using inverse Finite Element Method. Ocean Eng..

[27] Zhao F., Xu L., Bao H., Du J. (2020). Shape sensing of variable cross-section beam using the inverse finite element method and isogeometric analysis. Measurement.

[28] Kefal A., Tabrizi I.E., Yildiz M., Tessler A. (2021). A smoothed iFEM approach for efficient shape-sensing applications: Numerical and experimental validation on composite structures. Mech. Syst. Signal Process..

[29] Oboe D., Colombo L., Sbarufatti C., Giglio M. (2021). Comparison of strain pre-extrapolation techniques for shape and strain sensing by iFEM of a composite plate subjected to compression buckling. Compos. Struct..

[30] Kefal A., Oterkus E., Tessler A., Spangler J.L. (2016). A quadrilateral inverse-shell element with drilling degrees of freedom for shape sensing and structural health monitoring. Eng. Sci. Technol. Int. J..

[31] Kefal A., Oterkus E. (2016). Displacement and stress monitoring of a chemical tanker based on inverse finite element method. Ocean Eng..

[32] Kefal A., Oterkus E. (2016). Displacement and stress monitoring of a Panamax containership using inverse finite element method. Ocean Eng..

[33] Kefal A., Mayang J.B., Oterkus E., Yildiz M. (2018). Three-dimensional shape and stress monitoring of bulk carriers based on iFEM methodology. Ocean Eng..

[34] Kefal A. (2015). Structural Health Monitoring of Marine Structures by Using Inverse Finite Element Method. Ph.D. Thesis.

[35] Li M., Kefal A., Oterkus E., Oterkus S. (2020). Structural health monitoring of an offshore wind turbine tower using iFEM methodology. Ocean Eng..

[36] Esposito M., Gherlone M. (2020). Composite wing box deformed-shape reconstruction based on measured strains: Optimization and comparison of existing approaches. Aerosp. Sci. Technol..

[37] Foss G., Haugse E. Using modal test results to develop strain to displacement transformations. Proceedings of the 13th International Modal Analysis Conference.

[38] Ko W.L., Richards W.L., Tran V.T. (2007). Displacement Theories for In-Flight Deformed Shape Predictions of Aerospace Structures.

[39] Cortés A., Romate X.F., Jiménez-Suárez A., Campo M., Prolongo M.G., Ureña A., Prolongo S.G. (2020). 3D printed anti-icing and de-icing system based on CNT/GNP doped epoxy composites with self-curing and structural health monitoring capabilities. Smart Mater. Struct..

[40] Lammering R., Gabbert U., Sinapius M., Schuster T., Wierach P. (2017). Lamb-Wave Based Structural Health Monitoring in Polymer Composites.

[41] Fu H., Khodaei Z.S., Aliabadi M.F. (2018). An event-triggered energy-efficient wireless structural health monitoring system for impact detection in composite airframes. IEEE Internet Things J..

[42] Cerracchio P., Gherlone M., Tessler A. (2015). Real-time displacement monitoring of a composite stiffened panel subjected to mechanical and thermal loads. Meccanica.

[43] Kefal A., Tabrizi I.E., Tansan M., Kisa E., Yildiz M. (2021). An experimental implementation of inverse finite element method for real-time shape and strain sensing of composite and sandwich structures. Compos. Struct..

[44] Cerracchio P., Gherlone M., Di Sciuva M., Tessler A. (2015). A novel approach for displacement and stress monitoring of sandwich structures based on the inverse Finite Element Method. Compos. Struct..

[45] Kefal A., Tessler A., Oterkus E. (2017). An enhanced inverse finite element method for displacement and stress monitoring of multilayered composite and sandwich structures. Compos. Struct..

[46] Tessler A., Di Sciuva M., Gherlone M. (2010). A consistent refinement of first-order shear deformation theory for laminated composite and sandwich plates using improved zigzag kinematics. J. Mech. Mater. Struct..

[47] Gherlone M., Cerracchio P., Mattone M., Di Sciuva M., Tessler A. (2012). Shape sensing of 3D frame structures using an inverse finite element method. Int. J. Solids Struct..

[48] Roy R., Gherlone M., Surace C. (2021). A shape sensing methodology for beams with generic cross-sections: Application to airfoil beams. Aerosp. Sci. Technol..

[49] Kefal A., Oterkus E. Shape sensing of aerospace structures by coupling isogeometric analysis and inverse finite element method. Proceedings of the 58th AIAA/ASCE/AHS/ASC Structures, Structural Dynamics and Materials Conference.

[50] Papa U., Russo S., Lamboglia A., Del Core G., Iannuzzo G. (2017). Health structure monitoring for the design of an innovative UAS fixed wing through inverse finite element method (iFEM). Aerosp. Sci. Technol..

[51] Kefal A., Yildiz M. (2017). Modeling of sensor placement strategy for shape sensing and structural health monitoring of a wing-shaped sandwich panel using inverse finite element method. Sensors.

[52] Kammer D.C., Tinker M.L. (2004). Optimal placement of triaxial accelerometers for modal vibration tests. Mech. Syst. Signal Process..

[53] Kammer D.C. (1991). Sensor placement for on-orbit modal identification and correlation of large space structures. J. Guid. Control. Dyn..

[54] Salama M., Rose T., Garba J. Optimal placement of excitations and sensors for verification of large dynamical systems. Proceedings of the 28th Structures, Structural Dynamics and Materials Conference.

[55] Yi T.H., Li H.N., Gu M. (2011). Optimal sensor placement for structural health monitoring based on multiple optimization strategies. Struct. Des. Tall Spec. Build..

[56] Ostachowicz W., Soman R., Malinowski P. (2019). Optimization of sensor placement for structural health monitoring: A review. Struct. Health Monit..

[57] Yang W., Yang H., Tang S. (2019). Optimization, and control application of sensor placement in aero servo elastic of UAV. Aerosp. Sci. Technol..

[58] Kefal A. (2019). An efficient curved inverse-shell element for shape sensing and structural health monitoring of cylindrical marine structures. Ocean Eng..

[59] Abdollahzadeh M.A., Tabrizi I.E., Kefal A., Yildiz M. (2021). A combined experimental/numerical study on deformation sensing of sandwich structures through inverse analysis of pre-extrapolated strain measurements. Measurement.

[60] Kefal A., Oterkus E. (2020). Isogeometric iFEM analysis of thin shell structures. Sensors.

[61] Roy R., Tessler A., Surace C., Gherlone M. (2020). Shape sensing of plate structures using the inverse finite element method: Investigation of efficient strain-sensor patterns. Sensors.

[62] Cook R.D. (1994). Four-node ‘flat’ shell element: Drilling degrees of freedom, membrane-bending coupling, warped geometry, and behavior. Comput. Struct..

[63] Tessler A., Hughes T.J. (1983). An improved treatment of transverse shear in the Mindlin-type four-node quadrilateral element. Comput. Methods Appl. Mech. Eng..

[64] Bentley P.J., Wakefield J.P. Hierarchical crossover in genetic algorithms. Proceedings of the 1st On-Line Workshop on Soft Computing (WSC1).

[65] Liu W., Gao W.C., Sun Y., Xu M.J. (2008). Optimal sensor placement for spatial lattice structure based on genetic algorithms. J. Sound Vib..

[66] Guo H.Y., Zhang L., Zhang L.L., Zhou J.X. (2004). Optimal placement of sensors for structural health monitoring using improved genetic algorithms. Smart Mater. Struct..

[67] Swann C., Chattopadhyay A. (2006). Optimization of piezoelectric sensor location for delamination detection in composite laminates. Eng. Optim..

